# Recent advances in the structural diversity of reaction centers

**DOI:** 10.1007/s11120-021-00857-9

**Published:** 2021-06-26

**Authors:** Christopher J. Gisriel, Chihiro Azai, Tanai Cardona

**Affiliations:** 1 Department of Chemistry, Yale University, New Haven, CT 06520 USA; 2 College of Life Sciences, Ritsumeikan University, Kusatsu, 525-8577 Japan; 3 Graduate School of Life Sciences, Ritsumeikan University, Kusatsu, 525-8577 Japan; 4 Department of Life Sciences, Imperial College London, London, UK

**Keywords:** Photosynthesis, Reaction center, Green sulfur bacteria, Evolution, Cryo-EM

## Abstract

Photosynthetic reaction centers (RC) catalyze the conversion of light to chemical energy that supports life on Earth, but they exhibit substantial diversity among different phyla. This is exemplified in a recent structure of the RC from an anoxygenic green sulfur bacterium (GsbRC) which has characteristics that may challenge the canonical view of RC classification. The GsbRC structure is analyzed and compared with other RCs, and the observations reveal important but unstudied research directions that are vital for disentangling RC evolution and diversity. Namely, (1) common themes of electron donation implicate a Ca^2+^ site whose role is unknown; (2) a previously unidentified lipid molecule with unclear functional significance is involved in the axial ligation of a cofactor in the electron transfer chain; (3) the GsbRC features surprising structural similarities with the distantly-related photosystem II; and (4) a structural basis for energy quenching in the GsbRC can be gleaned that exemplifies the importance of how exposure to oxygen has shaped the evolution of RCs. The analysis highlights these novel avenues of research that are critical for revealing evolutionary relationships that underpin the great diversity observed in extant RCs.

**Supplementary Information:**

The online version contains supplementary material available at 10.1007/s11120-021-00857-9.

## Introduction

Reaction center (RC) proteins are at the heart of photosynthesis. Within a single protein complex, light excites antenna pigments and energy is transferred to the electron transfer (ET) domain of the RC where charge separation results in the transfer of electrons through a series of cofactors arranged in a gradient of potential energy (Blankenship 2008). Electrons are donated to cofactors that diffuse away from the RC, reducing equivalents that fuel metabolism, and electron donors reduce the oxidized RC to poise it for another charge separation event. This broad description holds true for all RCs, but extreme diversity is found among RCs from different organisms, a result of natural selection in a given ecological niche and a long evolutionary history spanning billions of years. For classification purposes, RCs have traditionally been divided into two different types, type I and type II, that are defined by the molecular identity of the electron acceptors within the protein complex. RCs whose terminal electron acceptor is a [4Fe–4S] cluster are termed type I (Vassiliev et al. 2001), whereas RCs whose terminal electron acceptor is a quinone molecule are termed type II (Diner et al. 1991). In oxygenic photosynthesis both the type I and type II RCs, called photosystem I (PSI) and photosystem II (PSII), function in series. Anoxygenic phototrophs contain only a single type of RC, either type I or type II, which we refer to herein as RC1 and RC2, respectively.

The first RC structure to be solved was an RC2 from the purple bacterium *Blastochloris viridis* (PbRC) in 1984 (Deisenhofer and Michel 2005). The structures of PSI and PSII, both from the cyanobacterium *Thermosynechococcus elongatus*, were solved in 2001 (Fromme et al. 2001; Zouni et al. 2001). Finally, in 2017, a representative structure of an RC1 was solved from *Heliobacterium modesticaldum* (HbRC) (Gisriel et al. 2017). This allowed for the comparison of structures from all four RC classes (Orf et al. 2018a) and important evolutionary relationships were and continue to be discovered. A challenge, however, is that unlike PSI and PSII, RCs within a class involved in anoxygenic photosynthesis (RC1 or RC2) differ greatly. Recent advances in cryo-electron microscopy (cryo-EM) have led to an expanding collection of molecular structures of RC complexes appearing in the literature: most recently, that of a second RC1 representative, the RC from the green sulfur bacterium (GsbRC), *Chlorobaculum tepidum* (Chen et al. 2020). Despite the low sequence identity with other RCs, the GsbRC maintains the common transmembrane core domain motif (Sadekar et al. 2006) that coordinates cofactors for ET but also exhibits unique antenna composition, subunit positions, and interactions with the peripheral antenna. The new GsbRC structure allows for comparisons that provide insights into the evolution of RCs and predictive power for understanding RCs whose molecular structures have not yet been resolved.

Here, we analyze the GsbRC structure and place the observations in the context of structural analyses of other RCs. These observations are used to describe general themes and important differences in RC characteristics such as electron donation mechanisms, specificity for ET chain cofactors, and antenna pigment arrangement. On the basis of these structural comparisons, we predict features of representative RC1 structures from the phyla Acidobacteria and Chloroflexi and point out important research directions for each RC complex. We then bridge the gap between structural comparisons and sequence-based phylogenetic analyses allowing us to provide insight into the evolution of photosynthesis that has shaped life on Earth.

## An overview of the structure and evolution of reaction centers

**Fig. 1:**
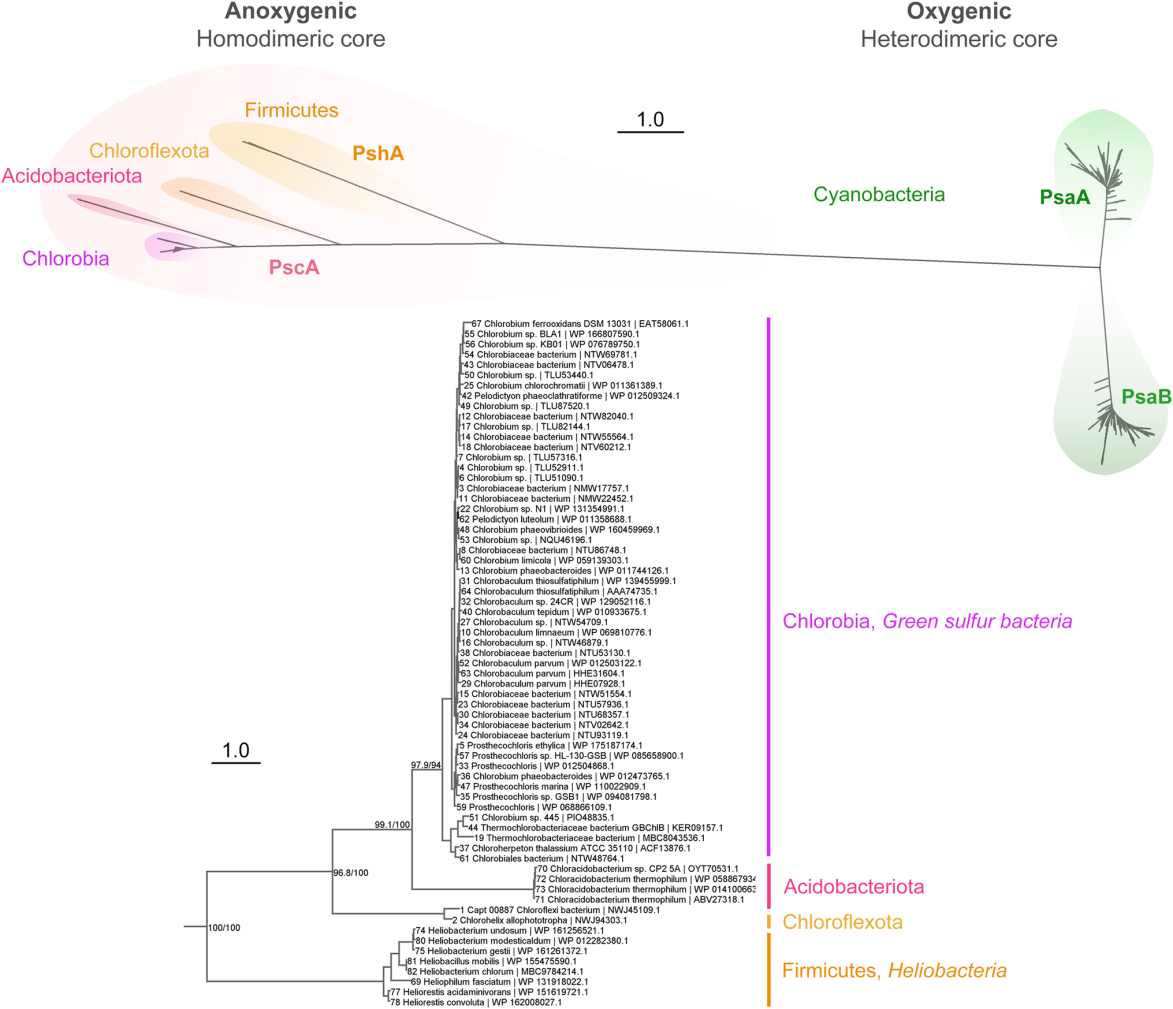
Complete Maximum Likelihood phylogeny of type I RCs. The top tree was calculated using sequences from all type I RCs. Sequences were retrieved from National Center for Biotechnology Information (NCBI) RefSeq (O’Leary 2016), aligned with Clustal Omega (Sievers 2011), and phylogenies were built with IQ-TREE V.2.1.2 (Minh 2020). The top tree is characterized by a long branch separating the homodimeric RC1 from PSI used in oxygenic photosynthesis. Long branches show the large evolutionary distance between the different classes. The bottom tree zooms in on the homodimeric RC1 sequences and it is rooted using cyanobacterial PSI, which is not shown for clarity. Taxonomic names follow the proposed nomenclature by the Genome Taxonomy Database (Parks 2020). Scale bars represent the number of substitutions per site

The structure of the GsbRC fills an important gap in our understanding of photosystem function and evolution, not only because of its key position in the tree of life (Fig. 1), but also because of the historical significance of the green sulfur bacteria as one of the first isolated and described models for the study of bacterial photosynthesis (Swarthoff and Amesz 1979). It is well accepted that photosynthesis originated over 3.4 billion years ago (Tice and Lowe 2004; Fischer et al. 2016) and that RCs evolved with a homodimeric core (Blankenship 1992, 2010; Olson and Blankenship 2004). Thus, the homodimeric GsbRC may not only present novel adaptations unique to its group, but it may also retain ancestral traits tracing to the dawn of life on Earth, some of which may predict characteristics of photosynthetic life on other planets (Lingam and Loeb 2020; Beatty 2005).

All RCs exhibit an ET domain comprised of five transmembrane helices (TMH) from each of two subunits, therefore 10 TMH in total, that coordinate the ET chain cofactors between them. Of the four classes of RCs, only RC1 feature *C*_2_ symmetry at the core. For the other three RC classes (PSII, PSI, and RC2) the core has evolved structural and functional asymmetry, resulting in pseudo-*C*_2_ symmetry. Heterodimerization in type II RCs is rationalized to have arisen to establish the two-electron gate (Velthuys and Amesz 1974). In PSII, heterodimerization has additionally been linked to the development of water oxidation (Cardona et al. 2019). In PSI, it has been proposed that heterodimerization evolved as a protective mechanism to minimize the production of reactive oxygen species (Orf et al. 2018a; Rutherford et al. 2012). The type I RCs and PSII exhibit six additional TMH on each side of the ET domain that coordinate antenna pigments; therefore, the core of the RC complexes from all known RCs except RC2 is comprised of 22 TMH. For reference, the core of the GsbRC and HbRC and their respective domains are shown in Supplementary Fig. 1.

The phylogeny of type I RCs is characterized by a deep dichotomy that separates RC1 from PSI (Fig. 1 and Supplementary Fig. 2). Among the homodimers, the HbRC makes a lineage that is sister to a larger group that includes the recently discovered sequences from *Candidatus* Chlorohelix allophototropha (CfxRC1) (Tsuji et al. 2020), the GsbRC, and the RC of *Chloracidobacterium thermophilum* (CabRC) (Tank and Bryant 2015), the latter being the only described phototrophic member of the Acidobacteria. The availability of structural information allows these relationships to be further validated using structure-based sequence alignments (Supplementary Fig. 2) that are also in agreement with taxa-rich phylogenies (Fig. 1). Structures of the CabRC and CfxRC1 have not yet been elucidated but the GsbRC structure enhances the reliability of structural predictions based on sequence homology.

The contrasting level of divergence in PSI and RC1, which is low in the former and high in the latter, reflects differences in selective pressure for their structural evolution. Though oxygenic phototrophs have diverged widely over billions of years, the core structure and function of PSI have remained the same, suggesting substantial selective pressure against mutations that cause functional changes through speciation. Conversely, the high level of divergence in RC1s suggests less selective pressure; the large phylogenetic distances among RC1s, when taken together with their multi-billion year history and slow rates of change (Cardona 2018; Oliver et al. 2021) hint to the possibility that the diversity of extinct, but also extant phototrophs, is largely underestimated. It is plausible that other groups with novel phototrophs and type I RCs will continue to be discovered.

## Comparison of the GsbRC to other RCs

The recent GsbRC structure provides the first view of its unique architecture (Chen 2020). The homodimeric core coordinates ET cofactors that are positioned similarly to those observed in the HbRC. The main pigment found in the GsbRC is bacteriochlorophyll (BChl) *a*, but four Chl *a* molecules are found in the ET chain. Of the known type I RC structures, the GsbRC exhibits the fewest antenna bacteriochlorophylls or chlorophylls (hereafter collectively referred to as “(B)Chls”). The long-studied Fenna–Matthews–Olson peripheral antenna protein (FMO) (Olson 2004) is bound to the antenna domain of the GsbRC core, providing insight into its role in energy transfer. We note that in our comparison below, we adopt the ET chain nomenclature used in the GsbRC publication (Chen 2020) which is consistent with that used in the publications of both the first PSI (Jordan, 2001) and HbRC (Gisriel 2017) structures (Supplementary Fig. 1).

### Electron donation and donor-side surface characteristics

#### Electron donors to RCs

Due to the diversity of electron donors and cellular architecture, the donor-side surface of each RC is variable. The GsbRC is known to contain two permanently bound transmembrane subunits called PscC, each with a soluble cytochrome domain, cyt *c*_z_, that extends into the periplasm on the donor side to reduce the primary electron donor, P_840_ following its oxidation (Oh-oka et al. 1995, 1997; Tsukatani et al. 2008; Hirano 2010). The GsbRC structure lacks the PscC subunits, presumably due to their loss during sample preparation, so visualizing the orientation of cyt *c*_z_ on the donor surface is challenging, though some interacting residues have been proposed by cross-linking (He et al. 2014). The HbRC is known to be reduced by cyt *c*_553_ which is tethered to the membrane via a covalently bound lipid (Albert et al. 1998; Kashey et al. 2014; Prince et al. 1985). Though cyt *c*_z_ and cyt *c*_553_ are kept in proximity to their respective RCs differently, they nevertheless appear to have similar modes of electron donation. Unlike the permanently bound tetraheme subunit observed in most RC2s, electron donation from cyt *c*_z_ to P_840_^+^ in the GsbRC is dependent upon viscosity (Oh-oka et al. 1997; Franken and Amesz 1997), making it similar to the membrane-tethered cyt *c*_553_ that donates electrons to the HbRC.

The electrostatic surfaces calculated from the GsbRC and HbRC structures exhibit negatively charged patches adjacent to the center of the complex (Supplementary Fig. 3). The electrostatic surface calculated from the structure of cyt *c*_z_ and a cyt *c*_553_ homology model both exhibit positively charged faces near the surface-exposed edge of heme pyrrole ring II (Hirano 2010) that probably binds to the negatively charged regions on the RC surface, suggesting that upon donor binding, the distance between the heme and P in both cases is similar to that observed by cyt *c*_2_ in the PbRC structure (Axelrod 2002). PSI is reduced by plastocyanin (Hippler et al. 1997) and cyt *c*_6_ whose binding are driven by a combination of hydrophobic and electrostatic interactions (Sommer et al. 2006). Unlike the donors to the HbRC and GsbRC, plastocyanin and cyt *c*_6_ are not permanently bound near the RC core. This difference may be explained by the cellular architecture: PSI is found within thylakoid membranes that probably regulate the concentration of electron donors but neither green sulfur bacteria or heliobacteria exhibit thylakoid membranes. Additionally, the mode of electron donation in oxygenic photosynthesis may be shaped by the requirement to shuttle electrons between PSII and PSI via the cyt *b*_6_*f* complex.

Despite these differences, a commonality among type I RCs appears to be that all known electron donors to P are at least partially diffusible in contrast to type II RCs which are not, save a few exceptions where the bound cytochrome subunit is thought to have been lost during evolution (Matsuura 1994; Tsukatani 2004; Matsuura and Shimada 1990; Nitschke and Dracheva 1995). Neither of the genomes from organisms containing the CabRC or CfxRC1 contain PscC-like genes, therefore their mode of RC reduction is unclear; however, He et al. showed that monoheme *c*-type cytochromes co-purify with the CabRC (He 2019) which may serve a function similarly to that of PscC in the GsbRC or cyt *c*_553_ to the HbRC. Homology models of the CabRC and CfxRC1 exhibit low sequence identity, especially for the CabRC where large insertions are present in looping regions on the donor side. It is of interest to understand the unique surface features that govern electron donation to the CabRC and CfxRC1 in future work.

#### P-helices and Ca^2+^ site

Positioned directly toward the donor surface relative to P in all RCs are two surface helices, one from each of the core polypeptides, termed the “P-helices” (Orf et al. 2018a) (Supplementary Fig. 4). In PSI from *Chlamydomonas reinhardtii*, a Trp residue in each P-helix which is conserved in all PSI core polypeptides was shown to be important for ET from plastocyanin and cyt *c*_6_ (Sommer et al. 2004; Hippler and Drepper 2006). This Trp is conserved in the GsbRC structure and sequence, and the CabRC sequence, suggesting that the reduction of P^+^ from each of these RCs may occur similarly to that by which P_700_^+^ is reduced in PSI. In the HbRC, however, this Trp residue is instead an Ile which is also conserved as Ile in the CfxRC1. A mechanism of P^+^ reduction other than that involving the conserved Trp residue has not been proposed, but future site-directed mutagenesis experiments may help to understand which residues near P in the HbRC and CfxRC1 may be involved in the reduction of P^+^.

The crystal structure of the HbRC revealed a Ca^2+^-binding site adjacent to the P-helix on each core subunit at a position similar to that of the redox-active D1-Tyr_Z_ and D2-Tyr_D_ of PSII (Supplementary Fig. 5). Cardona and Rutherford described structural similarities between this Ca^2+^-binding site and the Mn_4_CaO_5_ cluster of PSII (Umena et al. 2011), suggesting a plausible mechanism for the evolution of water-splitting from structural elements thought to be present in the earliest RCs (Cardona and Rutherford 2019). Based on sequence similarity it was predicted that the GsbRC exhibited a similar Ca^2+^-binding site, which was confirmed by the cryo-EM structure (Chen 2020). There are, however, notable differences between the RCs as described in Supplementary Fig. 5. In particular, the GsbRC Ca^2+^-binding site appears less exposed to the cytosolic medium. The CabRC is also likely to possess an analogous Ca^2+^-binding site because all coordinating residues are conserved relative to the PscA sequence from the GsbRC. In contrast, the CfxRC1 is unlikely to have the Ca^2+^ site as the residues at homologous positions contain mostly sidechains that cannot serve as ligands, which may suggest different modes of P^+^ reduction if the Ca^2+^ is involved.

The role of this Ca^2+^ site is unknown, but its presence in several distantly-related RC1s may indicate that this was a feature of the earliest RC1. It is possible that this Ca^2+^ plays a structural role in the configuration of the electron-donor docking site or provides greater stability against thermal denaturation. ET experiments replacing this ion with other divalent metals could indicate whether the site tunes the kinetics of P^+^ reduction. In fact, in isolated membranes of *Heliobacterium gestii* it was observed that the addition of Mg^2+^ restored a purification-induced slowdown of P^+^ reduction kinetics (Oh-oka et al. 2002). The site highlights several residues that could be important for ET, particularly a conserved PscA-Tyr599 adjacent to the Ca^2+^ (Supplementary Fig. 5). Given the structural parallels with the water-splitting cluster of PSII, elucidating the function of this site may give clues on the earliest stages of the origin of water-splitting in oxygenic photosynthesis.

#### Themes of electron donation to the RCs

**Fig. 2:**
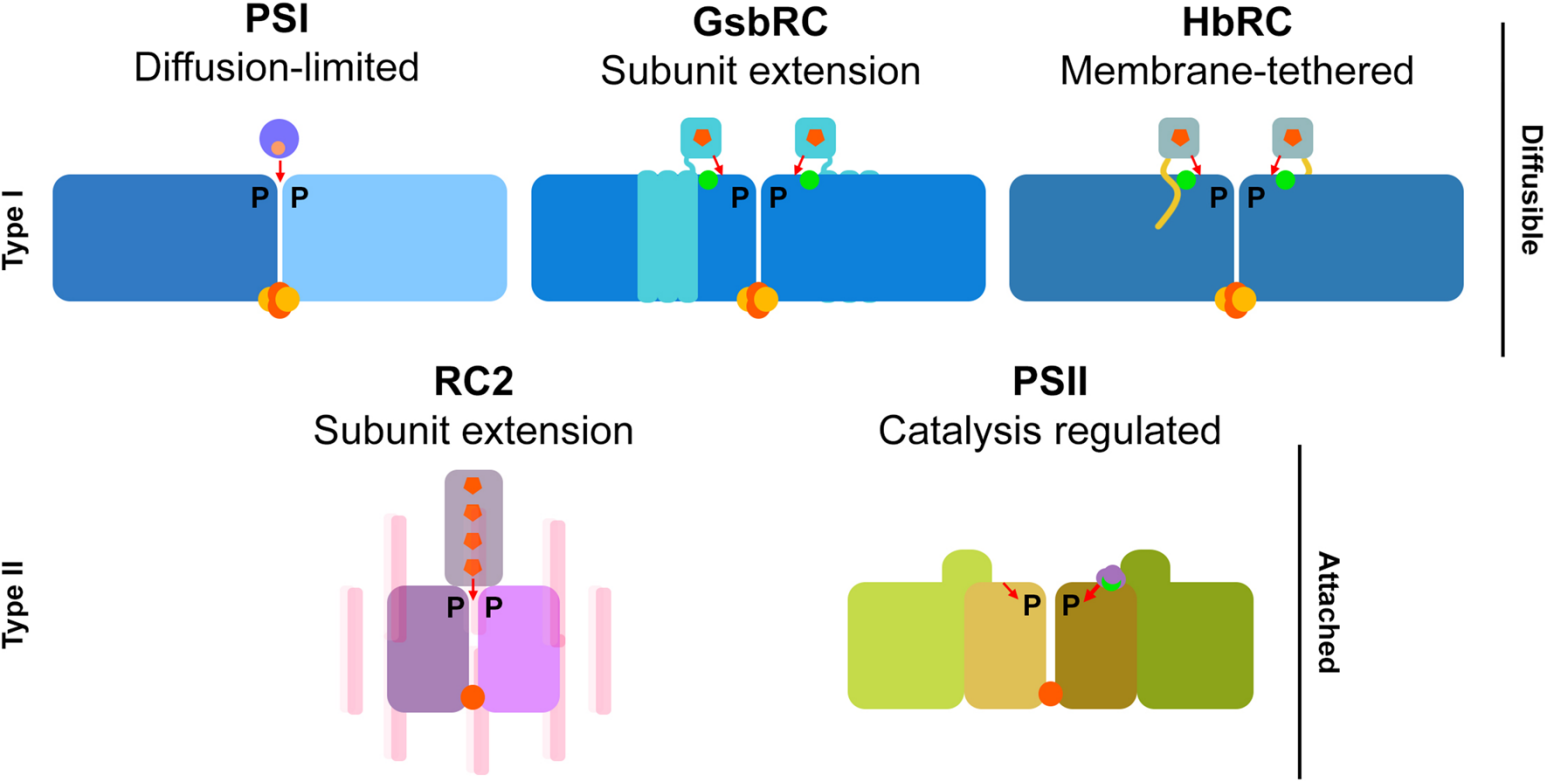
Themes of electron donation in RCs. Modes of electron donation and directionality (red arrows) are shown. Type I RCs (top row) exhibit only diffusible electron donors and type II RCs exhibit bound electron donors. Ca^2+^ sites are shown with green spots. Hemes are shown as orange pentagons. Core-associated [4Fe-4S] clusters are show as orange and yellow spheres. Non-heme irons are shown as single orange spheres. A tetraheme cytochrome is commonly found in anoxygenic RC2s, however, cases in which this subunit has been lost are known within the Proteobacteria

The donor-side structural details highlight common themes that are observed when comparing the donor interactions of the RCs (Fig. 2). First, electron donation to the RCs is observed as diffusion-limited, membrane-tethered, catalysis-regulated, or as a subunit extension. Second, the presence of the Ca^2+^ site seems to be correlated with uni- or bi-directional reduction of P^+^. In PSII, though the main pathway of P^+^ reduction is via Tyr_Z_ and the Mn_4_CaO_5_ cluster, Tyr_D_ has several important roles in photoprotection and assembly, which are not yet entirely understood (Rutherford et al. 2004; Styring et al. 2012). In contrast, PSI and RC2 are reduced from a single binding site, the former of which was recently confirmed by a cryo-EM structure with plastocyanin bound (Caspy et al. 2020). The site specificity for binding cyt *c*_6_ to the PSI donor surface is unclear, but probably exhibits a single binding site like that of plastocyanin. In the RC2, a single heme-containing subunit is bound to reduce P^+^, although this has been lost within the Proteobacteria in a few cases (Matsuura 1994; Tsukatani 2004; Matsuura and Shimada 1990; Nitschke and Dracheva 1995). Studies of site-directed mutants that aim to understand donor binding, molecular structures of the CabRC and CfxRC1, and a more complete structure of the GsbRC will be required to better understand the role of the Ca^2+^ site, C-terminal extensions, P-helix interactions, and modes of donor binding in RC function and evolution.

### Electron transfer chain

#### Membrane thickness

The ET cofactors span the membrane from the donor side to the acceptor side, allowing electrons to traverse the lipid bilayer. The predicted hydrophobic thickness calculated by the Positioning of Proteins in Membranes (PPM) server (Lomize et al. 2012) for the GsbRC is similar to other RCs, ~ 30 Å, except that from the HbRC which is ~ 3–4 Å shorter than the others (Table S1). Unlike PSI, a quinone is not required for forward ET to F_X_ in the HbRC (Kleinherenbrink et al. 1993) or GsbRC (Hager-Braun et al. 1997); however, the HbRC has been shown to terminally reduce menaquinone to menaquinol (Kashey et al. 2018). Upon the elucidation of the HbRC structure (Gisriel 2017), it was observed that the distance between P_800_ and F_X_ is shorter than that for PSI. An evolutionary hypothesis derived from this observation is that this arrangement may be a vestige of an ancient RC ancestor that could reduce either a [4Fe–4S] cluster or a quinone (Orf et al. 2018a). Indeed, such a RC ancestor has been suggested previously (Sadekar et al. 2006; Rutherford 1996; Allen 2005; Orf et al. 2018b). An alternative hypothesis is that such an arrangement is unique to heliobacteria: lateral gene transfer of the photosynthetic gene cluster from an organism with a membrane thickness ~ 30 Å to a heliobacterial ancestor with a shorter membrane thickness applied pressure to shorten the ET chain, causing peculiar ET characteristics in the HbRC. The latter hypothesis implicates higher-order biological attributes in shaping the evolution of RCs. The former hypothesis is supported by the observation that the GsbRC ET cofactors are in nearly identical positions compared to those in the HbRC. It is likely that the ancestor of the HbRC and GsbRC exhibited ET cofactor positions as that observed in the two RCs today, but it is unclear whether the GsbRC or other RC1s can alternatively reduce quinones like that observed in the HbRC. If they do, it would be clear that the current type I/type II RC nomenclature is somewhat inadequate because the [4Fe–4S] cluster may not always serve as the terminal electron acceptor in extant RC1s.

#### The special pair, P

In all RCs, P is comprised of a pair of (B)Chl molecules that are axially coordinated by His sidechains. In the homodimeric RC1s, this is always a pair of identical BChl′ molecules, an epimer of BChl that exhibits reversed stereochemistry about the 13^2^ carbon of the tetrapyrrole ring. P_700_ of the heterodimeric PSI is comprised of one Chl *a* and one Chl *a*′. The 13^2^ methoxycarbonyl substituent of this Chl *a*′ is involved in a H-bonding network with a central water molecule and four amino acids from PsaA: the backbone carbonyl of PsaA-Gly739, and the sidechains of PsaA-Thr743, PsaA-Tyr603, and PsaA-Ser607 (Supplementary Fig. 6). It was suggested that the H-bond network results in asymmetric spin density over P_700_^+^ (Jordan et al. 2001; Saito and Ishikita 2011); however, no significant difference in optical or electronic properties is observed between the two Chl *a* epimers in organic solvents, so we still lack an understanding of why Chl *a*′ is used in the special pair of PSI.

The light-induced infrared difference spectrum and the comparatively low redox potential of P/P^+^ in the HbRC and GsbRC suggest that the special pair in RC1 complexes do not exhibit H-bonding (Noguchi 2010; Azai et al. 2016). Consistent with this, there is no apparent H-bonding near the 13^2^ substituent of BChl *g*′ of P_800_ in the HbRC (Orf et al. 2018a), an observation that is especially confident due to the high resolution of the X-ray crystallographic data. In the GsbRC, PscA-Thr685 is modeled in an orientation that suggests a weak H-bond to the 13^2^ substituent of P_840_ (Supplementary Fig. 6); however, the local resolution map of the GsbRC cryo-EM structure suggests that the ET cofactors are all found in regions of ~ 2.7-Å resolution which is not high enough to resolve water molecules or the orientation of the PscA-Thr685 sidechain (Supplementary Fig. 7). PscA-Thr685 is conserved in the HbRC (PshA-Thr598) but is more confidently modeled with its hydroxyl substituent directed away from the 13^2^ substituent, forming a weak H-bond with the backbone carbonyl of PshA-Phe594 which is partially conserved as PscA-Tyr681 in the GsbRC structure. Thus, due to the low confidence of the PscA-Thr685 orientation in the GsbRC structure, and the conservation between residues observed in the high-resolution HbRC structure, we suggest that the true orientation of PscA-Thr685 is pointed away from the 13^2^ methoxycarbonyl substituent of BChl *a*′ which argues against H-bonding interactions to the 13^2^ methoxycarbonyl substituents of P_840_ in the GsbRC. This supports the hypothesis that the ancestor of type I RCs exhibited a pair of (B)Chl′ molecules whose 13^2^ substituents exhibited no H-bonding interactions, though such an argument excludes considerations regarding the biosynthetic pathways for these pigments, and it is still unclear why the 13^2^ epimers are required at this position other than steric constraints (Orf et al. 2018a).

Homology models of the CabRC and CfxRC1 also feature the conserved Thr residue whose hydroxyl group is pointing away from the 13^2^ substituent of P_800_ in the HbRC. Molecular structures of the CabRC, and CfxRC1 at resolutions that allow for the confident identification of water molecules and sidechains are needed to confirm the absence of H-bonding to P. The CabRC, especially, is a desirable target for structural studies aimed at understanding the role of P because unlike its bulk Mg-BChl *a*, P_840_ in the CabRC is a pair of Zn-BChl *a*′ (He 2019; Tsukatani et al. 2012), making it the only known phototroph that naturally synthesizes both Mg-BChl *a* and Zn-BChl *a*. The reason for Zn-BChl *a*′ specificity for P_840_ is presently unclear.

#### Acc

The (B)Chl type of the Acc pigment in the GsbRC is assigned as Chl *a*, rather than the BChl *a* found as the bulk pigment. At the achieved resolution, Chl *a* and BChl *a* are difficult to distinguish, but the Acc assignment as Chl *a* is consistent with a previous spectroscopic study (Permentier 2000). In all other known RCs, the Acc position is occupied by the same (B)Chl type as is found in the bulk antenna, making the GsbRC unique in this regard if the assignment is correct. The C3 acetyl moiety of BChl *a* requires slightly more space and a more polar environment relative to Chl *a* which has a vinyl moiety at C3. Selectivity for Chl *a* in the Acc site of the GsbRC may be conferred by PscA-Met436 which is positioned near the substituent at the C3 position of the tetrapyrrole ring because it decreases the available space required to bind BChl *a* and results in a more hydrophobic environment (Supplementary Figs. 8A, B). This also implies that the CabRC and CfxRC1, which instead of the Met residue have Val and Gly, respectively, may contain BChl *a* rather than Chl *a* in the Acc positions, the former of which is consistent with the previous hypotheses (He 2019).

The GsbRC is the first RC structure with a (B)Chl where the axial ligand is a Lys sidechain, that of PscA-Lys553 (Supplementary Figs. 8C, D). In PSI, both Acc positions are axially coordinated by water molecules H-bonded to Asn sidechains. Whereas these PSI Asn residues are partly conserved as Gln in PshA of the HbRC, in the HbRC structure the electron density corresponding to a molecule providing axial ligation to Acc appeared larger than would be expected for a water, and though a water is modeled in the PDB file, the authors suggested that it could be an ion (Gisriel 2017). PscA-Lys553 in the GsbRC structure is conserved in both the CabRC and CfxRC1, implying they, too, have Acc axial coordination via Lys residues. The discussion can be extended to type II RCs where the analogous position in PSII is a Chl *a* coordinated by a water (Umena et al. 2011) and in RC2 is BChl *a* coordinated by His sidechains (Deisenhofer et al. 1995). In the latter, mutagenesis experiments on the PbRC have suggested that little redox potential difference is observed between His- and water-axial ligation, and it was instead suggested that the stability of the site is enhanced by His coordination (Fufina et al. 2019). Differences are also observed in the RC2 from green non-sulfur bacteria (Chloroflexi) where the Acc site is occupied by a bacteriopheophytin instead of BChl on the inactive side of the ET chain, and in the RC2 found in phototrophic members of candidate phylum Eremiobacterota where an Asp sidechain coordinates the BChl in the inactive branch (Ward et al. 2019). The impacts of these differences observed in axial ligation to Acc are presently unclear but in type I RCs they likely influence the redox potentials of the sites, the quantification of which is an interesting research direction for future study.

#### A_0_ and A_1_

The A_0_ site in the GsbRC is also modeled as Chl *a* based on spectroscopic results (Kobayashi 2000). As with Acc, A_0_ in the GsbRC exhibits a large hydrophobic residue near the C3 position, PscA-Phe625, that is not present in either the HbRC or PSI, suggesting that the Phe confers selectivity for Chl *a* rather than the bulk BChl *a* in this site (Supplementary Figs. 9A, B). The corresponding residue in PscA sequences from the CabRC and CfxRC1 are a Phe and Leu, respectively, also suggesting these sites are Chl *a*. For the CabRC, this is consistent with nuclear magnetic resonance results (Zill 2018). In PSI, A_0_ is also Chl *a*, but so are all the rest of the pigments in the structure therefore no selectivity is required for (B)Chl type. In the HbRC, A_0_ is a Chl *a* derivative exhibiting a hydroxyl moiety at its 8^1^ position (Meent 1991). In the HbRC structure (Gisriel 2017), the S configuration of this substituent is modeled, but it was shown previously that the R configuration is found in the HbRC (Mizoguchi 2005). In such an orientation, the hydroxyl group of this substituent may form an OH–π interaction with the sidechain of PshA-Phe548 to confer site specificity.

Based on these observations, it seems likely that a characteristic of all type I RCs is that Chl *a* is found in the A_0_ position, which would have also been a trait of the ancestor of type I RCs. One exception is the recently solved PSI structure of a Chl *d*-utilizing cyanobacterium *Acaryochloris marina* (Hamaguchi 2021; Xu 2021) whose A_0_ pigment is assigned as pheophytin *a*. Given that PSII also has a Chl *a-*derived pigment at this position it seems likely that the earliest RC also bound Chl *a* at A_0_. We note that this suggestion neglects (B)Chl biosynthesis evolution that is debated and unresolved (Cardona 2016; Granick 1965; Xiong et al. 2000; Sousa et al. 2013; Lockhart et al. 1996; Blankenship et al. 2007).

In PSI and the HbRC, a H-bond is donated from an amino acid sidechain to the 13^1^-keto oxygen of A_0_ which is thought to be important for redox tuning (Müller et al. 2010; Li 2006). This residue in the HbRC is PshA-Ser553. The GsbRC conserves this Ser in sequence and structure (Supplementary Fig. 9C, D), and both the CabRC and CfxRC1 also conserve this Ser in their PscA sequences. Thus, so far, in addition to all type I RCs conserving Chl *a* in the A_0_ site (with the one exception noted above), that Chl *a* always exhibits an H-bond to the 13^1^-keto oxygen. In the RC1s, this H-bond is always provided by a Ser residue. In PSII, the 13^1^-keto oxygen of pheophytin *a* is observed within H-bonding distance of a Gln or Glu on each side (Umena et al. 2011), but for bacteriopheophytin in the PbRC (Deisenhofer et al. 1995) and other RC2s, this interaction is only on the active side. These observations may suggest that the ancestor of all RCs exhibited an H-bond to the 13^1^-keto oxygen of the pigment in this site.

The A_0_ axial ligand in the GsbRC is unclear. In PSI, both A_0_ sites exhibit axial coordination from a Met sidechain. In the HbRC, A_0_ is coordinated by a water molecule involved in a H-bonding network with PshA-Tyr577 and PshA-Ser545 (Supplementary Fig. 10), the latter of which aligns in sequence with the axially coordinating Met residues in PsaA and PsaB of PSI. This PshA-Ser545 aligns with PscA-Ala629 in both sequence and structure in the GsbRC. In both the standard sequence alignment and structure-based sequence alignment, PshA-Tyr577 appears unique to the HbRC. In any case, neither the Met residues in PsaA or PsaB, nor the Ser and Tyr residues involved in H-bonding in the HbRC, are conserved in the GsbRC structure. A_0_ in the GsbRC is probably coordinated by water molecules, but they must be in a different configuration than those observed in the HbRC. The CabRC likely exhibits similar A_0_ coordination as the GsbRC as it lacks the Met and H-bonding residues involved in A_0_ axial coordination in PSI and the HbRC, respectively. The CfxRC1, however, conserves the Met residue with PsaA and PsaB, and thus probably also coordinates A_0_ via a Met sidechain.

**Fig. 3:**
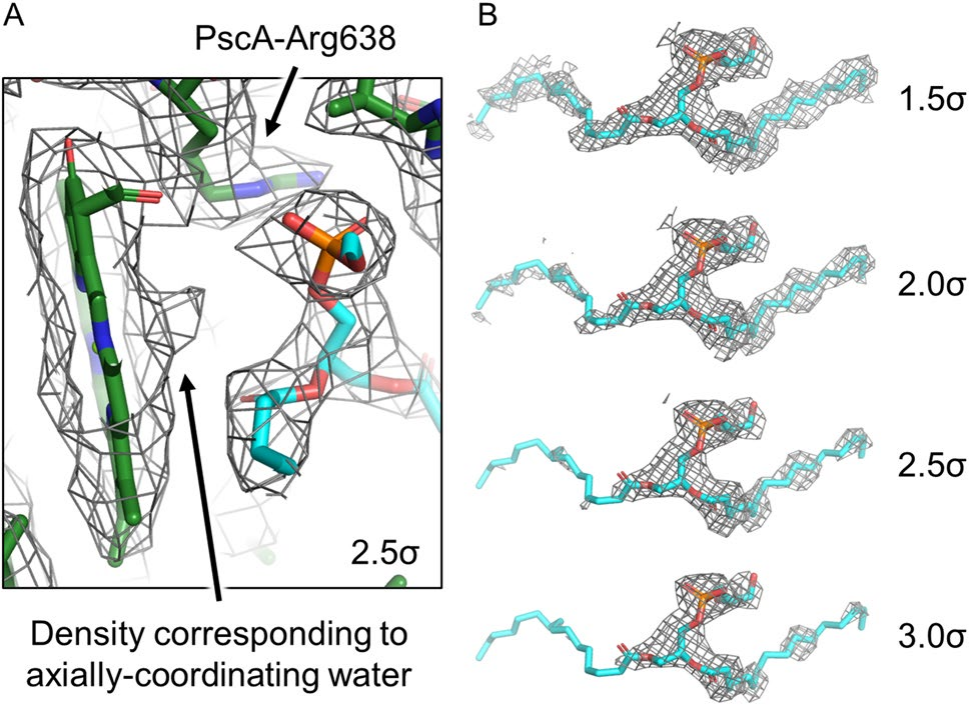
A_0_ coordination and unmodeled density in the A_1_ site. **A** shows the GsbRC model within its corresponding cryo-EM map at 2.5σ where a phosphatidylglycerol molecule (cyan) is fit into the nearby unmodeled density. The density corresponding to an unmodeled water that provides axial ligation to the central Mg of A_0_ and the location of PscA-Arg638 that provides a counter ion to the phosphatidylglycerol headgroup are labeled. **B** shows the unmodeled density near A_0_/A_1_ fit with a phosphatidylglycerol where the map is shown at four contour levels

The axial coordination of A_0_ of the GsbRC can be explained by density nearby the site analogous to A_1_ in PSI (Fig. 3). In the GsbRC cryo-EM map, density extends away from the central Mg of A_0_ toward the unmodeled density (Fig. 3A), reminiscent of that produced by a water molecule providing axial ligation. Though no water molecules are assigned in the 2.7-Å global resolution GsbRC structure, it may be possible to assign some high-occupancy water molecules as is exemplified by a recent 2.85-Å resolution cryo-EM structure of PSI from *T. elongatus* (Supplementary Fig. 11) (Kölsch 2020) which agrees with the X-ray crystal structure of PSI from the same organism (Jordan et al. 2001). The unmodeled density was described in the publication presenting the GsbRC structure and the authors attempted modeling in both a menquinone-7 and a Triton X-100 detergent molecule, but neither fit well (Chen 2020). We modeled a phosphatidylglycerol into this density which fit the profile nicely (Fig. 3B). The hydrocarbon tails of lipid molecules often feature high flexibility in experimental X-ray and cryo-EM maps which is consistent with the decrease in clear density near the tails as the contour level is increased. The anionic phosphate headgroup clearly provides a counter ion to PscA-Arg638, which is conserved in all RC1s, and it is likely that a water molecule is coordinated between the phosphate group and the central Mg of A_0_ (Fig. 3). Thus, we propose that the axial ligand of A_0_ in the GsbRC structure is a water molecule coordinated by the headgroup of this phosphatidylglycerol molecule. The lipid headgroup is positioned between A_0_ and F_X_, which undoubtedly would impact ET. Because the negative charge of the phosphate group would make the reduction potential of A_0_ significantly lower, even charge separation is likely to be affected.

It is unclear if the phosphatidylglycerol is a product of protein purification or a biologically relevant characteristic of the GsbRC. Similar unassigned electron density is observed in the HbRC structure, but the density is better resolved and appears to have a phosphate headgroup with a single isoprenoid tail (Gisriel 2017). The fact that two distantly-related RCs have mysterious density between A_0_ and F_X_, both exhibiting a phosphate headgroup providing a counterion to a conserved Arg sidechain, and that both of these RCs do not require a quinone molecule to achieve forward ET to F_X_ unlike PSI, strongly suggests biological relevance. The location of the buried Arg sidechain in hydrophobic regions of the RC1 structures is surprising because Arg sidechains have the highest pK_a_ among the 20 amino acid residues and are therefore likely to be fully charged. The neutralization of the charge via the anionic phosphatidylglycerol headgroup allows for the uncommon position of the Arg sidechain within membrane, but the Arg sidechain is still unexpectedly close to A_0_ and the membrane interior, which are very hydrophobic. The A_0_ proximity and surface exposure are likely to have functional significance, perhaps implications in quinone reduction and exchange.

On the other hand, the PscC subunits are not present in the GsbRC cryo-EM structure and may compromise its native state. The loss of these subunits was unexpected as GsbRC preparations from *C. tepidum* always contain at least PscA and PscC (He et al. 2014), which may suggest that sample preparation damaged the GsbRC complex used for cryo-EM. GsbRC complexes from *P. aestuarii* also lack the PscC subunit (Permentier 2000; Vasmel et al. 1983; Schmidt et al. 2000), but the cytochrome in *P. aestuarii* binds very loosely unlike that from *C. tepidum*, and thus may not be comparable, which is consistent with the profound sequence differences between PscC from the two species (Azai et al. 2010). Besides the core subunit, PscC is the only other transmembrane subunit associated with the GsbRC. In the HbRC, the only other subunit is PshX which has a single TMH and resides at the periphery of the homodimeric structure near the interface of the symmetry axis of the core (Gisriel 2017). Small transmembrane subunits occupy this location in nearly all other RCs as well. If the missing PscC subunits in the GsbRC reside near where the PshX subunits are located in the HbRC, then at least part of the transmembrane domain of PscC would be positioned very near the phosphatidylglycerol and A_0_ sites on each side, raising the possibility of structural modification in that location. Though they are not required for ET to F_X_, menaquinones are thought to serve as an acceptor in the GsbRC (Hauska et al. 2001; Azai 2011; Nitschke et al. 1990), and this phosphatidylglycerol may simply replace the menaquinone upon removal of PscC.

It is presently unclear whether the removal of PscC impacts ET, and further structural and biochemical studies are required to better understand the identities of the unmodeled densities in the GsbRC and HbRC. This may also apply to the CabRC and CfxRC1 whose core polypeptides also conserve the Arg residue that provides the counter ion for the aforementioned phosphate headgroups.

#### Terminal [4Fe–4S] clusters and ferredoxin binding.

**Fig. 4:**
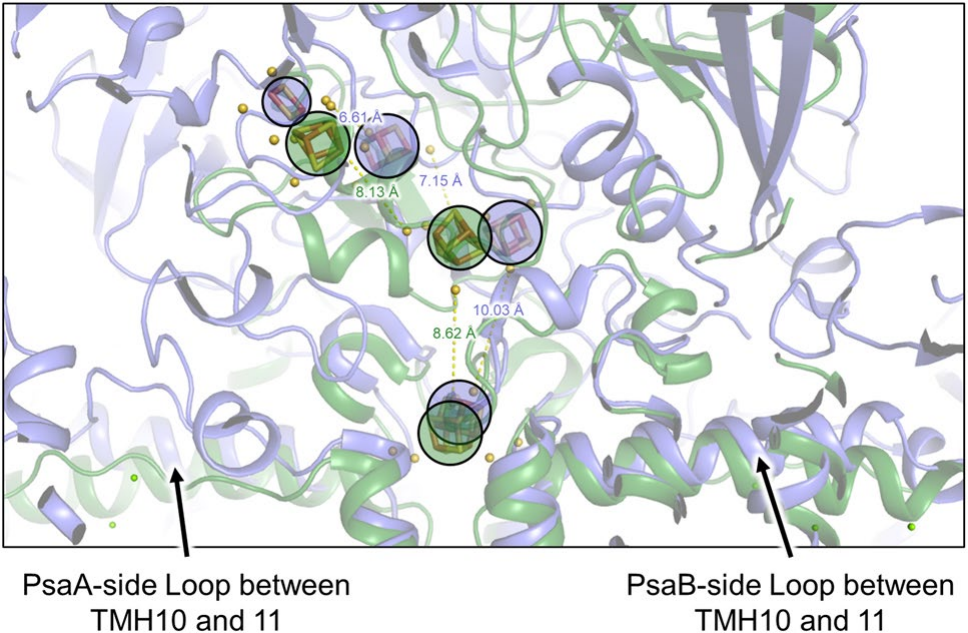
[4Fe–4S] clusters in the GsbRC and PSI. Structures of the GsbRC (green) and PSI (blue) are shown as transparent cartoons and the Fe–S clusters are additionally shown. The PSI structure is from *T. elongatus* with Fd bound (PDB 5ZF0). Fe–S clusters are circled and highlighted in the color corresponding to the structure and distances are shown between clusters including sulfur atoms of the coordinating cysteines (yellow spheres). Note that the 2Fe–2S cluster is from bound Fd in the PSI-Fd structure

The [4Fe-4S] clusters in the GsbRC are each coordinated by four Cys residues, a motif that is the hallmark of type I RCs. F_X_ is positioned in almost exactly the same location in the GsbRC compared to the HbRC (Chen 2020). Unlike the HbRC that only has a single [4Fe-4S] cluster bound at the core interface (Ferlez et al. 2016), both the GsbRC and PSI have an additional acceptor-side subunit, called PscB and PsaC, respectively, that coordinate two additional [4Fe-4S] clusters, F_A_ and F_B_. Aside from typical [4Fe–4S]-binding motifs, PscB and PsaC share little sequence identity (Figueras et al. 2002). Interestingly, a superposition of the structures shows that the positions of the three [4Fe–4S] clusters are similar between the GsbRC and PSI (Fig. 4). This suggests that the way in which the two RCs bind and reduce soluble ferredoxin (Fd) may also be similar. Fd was shown to bind in a cavity beside the stromal ridge of PsaC in PSI, placing F_B_ closest to the [2Fe–2S] cluster of Fd (Kubota-Kawai 2018) (Supplementary Fig. 12).

### Antenna

**Fig. 5:**
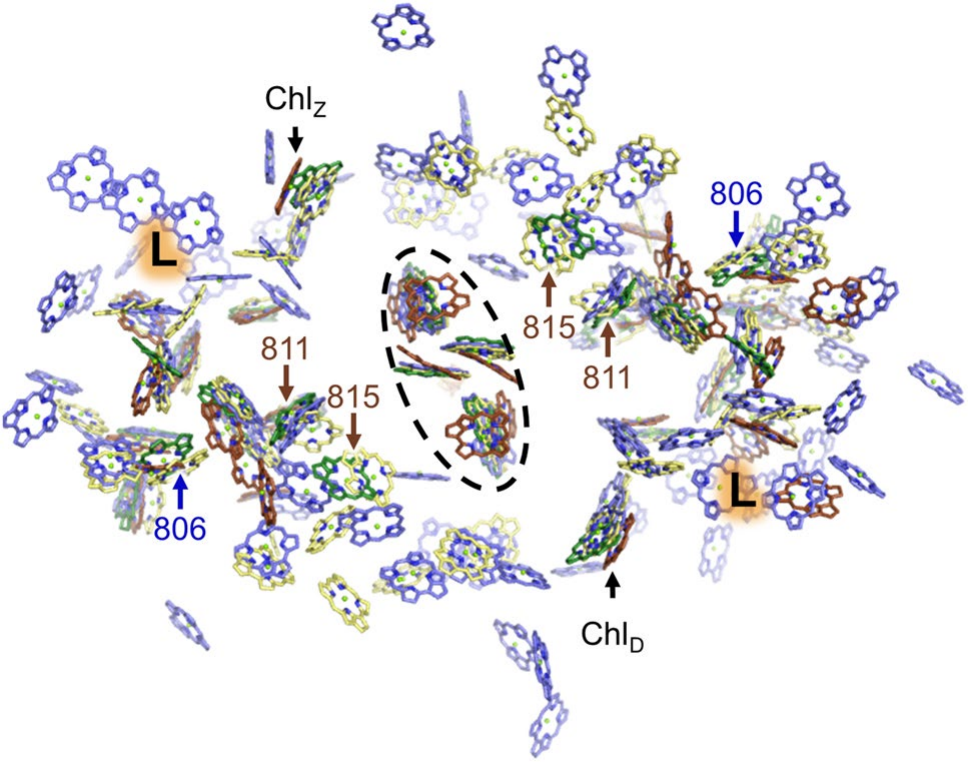
Antenna (B)Chl comparison. The tetrapyrrole rings are shown for (B)Chls in the GsbRC (green), HbRC (yellow), PSI (blue), and PSII (brown). The view is from the donor side and (B)Chls involved in the ET chain are circled by a dashed line. There is one GsbRC antenna BChl position found in the other RC structures except PSI (blue font and arrow). There are two GsbRC antenna BChl positions found in the other RC structures except PSII (brown font and arrows). The Chl_Z/D_ position conserved in all the RC structures is labeled. The “L” label indicates the lipid/carotenoid-containing region conserved in the GsbRC and PSII but not the HbRC or PSI

There are only 12 antenna BChls associated with each PscA monomer of the homodimeric GsbRC (Chen 2020). It is possible that the PscC subunits, which are missing from the GsbRC structure, bind additional antenna BChls; however, none of the three predicted transmembrane regions of PscC contain His residues. Because His residues in transmembrane regions provide ~ 70% of the (B)Chl axial coordination in other RC structures, their absence in the predicted TMH domain of PscC decreases the likelihood of the GsbRC binding additional antenna BChls, though this should be confirmed by future studies. Therefore, excluding those BChls found in the FMO trimer, the GsbRC likely binds only the 24 antenna BChl, all of which are present in the cryo-EM structure (Fig. 5). The homodimeric HbRC binds 25 antenna BChls per PshA and two BChls from each of two PshX subunits, therefore 54 antenna BChls in total, and cyanobacterial PSI binds 43 antenna Chls by PsaA, 38 antenna Chls by PsaB, and an additional nine antenna Chls by peripheral transmembrane subunits, therefore 90 antenna Chls in total. Thus, the number of antenna (B)Chls in the GsbRC is more similar to PSII where only 31 antenna Chls are bound in total (Umena et al. 2011). This suggests that PSII may retain features of type I RCs no longer seen in the PbRC or other RC2 complexes, which use an independently evolved antenna system, LH1 (Yu et al. 2018). These features shared by PSII and type I RCs are consistent with recent evidence suggesting an early origin of photosynthetic water oxidation (Cardona et al. 2019; Jabłońska and Tawfik 2021).

All of the 12 antenna BChl positions per core subunit in the GsbRC are also found in the HbRC and nearly all are conserved in PSI and PSII as well (Table S2). Such high structural similarity implies that the antenna (B)Chl sites in the GsbRC were present in the ancestor of all RCs. The three positions that are not conserved with PSI or PSII are all on the donor side of the protein, which is somewhat surprising because one might expect major antenna BChl differences on the acceptor side due to energy transfer requirements from the FMO BChls to the core. The GsbRC antenna position not found in PSI is on the periphery between TMH1, 2, and 3 (Fig. 5). The two GsbRC antenna positions not found in PSII are among the closest to the ETC and are located between TMH1, 10, and 11 (Fig. 5).

Though all 12 antenna BChls per PscA are conserved in the GsbRC relative to sites in the HbRC, the HbRC has 13 more BChls associated with its core polypeptide, PshA, that are not found in the GsbRC structure. The increased antenna BChl number in the HbRC may be a result of a trade-off of resources invested in peripheral antenna; whereas GsbRCs have chlorosomes for light harvesting, HbRCs have no peripheral antenna complexes and therefore rely upon the BChls bound to the RC complex for light harvesting. Of the 13 antenna BChls found in the HbRC but not the GsbRC, seven are on the donor side and six are on the acceptor side. Two of these are directly adjacent to the ET chain near the dimeric interface, one on each side of the membrane (orange circle in Supplementary Fig. 13). These are notably among the closest BChls to the ET chain in the HbRC, and are also nearby the Chl_Z/D_ sites of PSII that are conserved in all the RC structures except RC2. This suggests that energy transfer from the bulk antenna in the core to the ET chain is less well coupled in the GsbRC than it is in the HbRC and PSI.

**Fig. 6:**
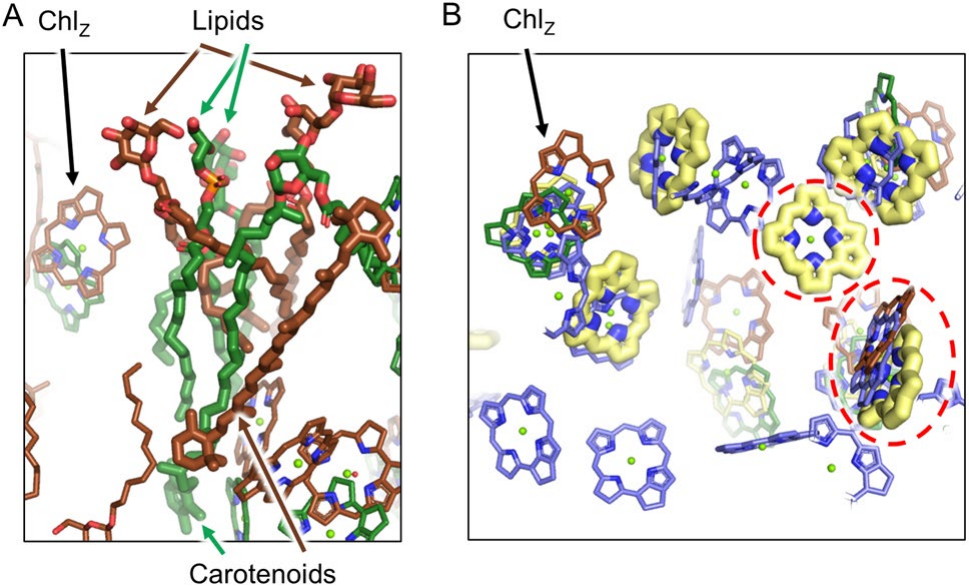
Peripheral lipid/carotenoid-containing region in the antenna domain of the GsbRC. **A** In both the GsbRC (green) and PSII (brown), two lipids and a carotenoid (sticks with large radii) are found in this location that correspond to the “L” labels in Fig. 5 and Fig. S13. **B** The same location, but showing only the tetrapyrrole rings from the GsbRC (green), HbRC (yellow), and PSI (blue). Those HbRC antenna BChls not found in the GsbRC structure in this region are shown with sticks of larger radii. The BChl sites in the HbRC not found in the GsbRC are conserved in PSI (but not in PSII), with only two exceptions that are circled with a red dashed line. The top is a site unique to the HbRC and the bottom is a site conserved in all the RCs, including PSII, except the GsbRC

Another notable difference is near the “L” in Fig. 5 and Supplementary Fig. 13 where five BChls are present in the HbRC but absent in the GsbRC (Fig. 6). In place of these five BChls, the GsbRC has a glycosylated carotenoid, “F39,” and two lipid molecules, the former of which is likely to be involved in energy quenching as described further below. Cofactors analogous to these are not observed in the HbRC or PSI, but two lipids and a β-carotene are found in PSII in approximately the same location (Fig. 6A). Therefore, in addition to the overall number of antenna BChls, this lipid and carotenoid arrangement in the GsbRC antenna domain also appears more similar to PSII than it does to other type I RCs. Furthermore, all but two of the 13 BChls present in the HbRC structure but not in the GsbRC structure are conserved with PSI but not PSII (Fig. 6B). Of the two that do not show this pattern, one is unique to the HbRC alone, suggesting that this site evolved after the HbRC antenna diverged from the others, and the other is conserved in the HbRC, PSI, and PSII, but not the GsbRC, suggesting that this site was lost in the latter or alternatively, that the site could have evolved independently three times.

Of the 12 antenna BChls modeled per PscA in the GsbRC structure, 11 have obvious axial ligands (Supplementary Fig. 14). Of these 11, the CabRC identically conserves 10 in the sequence alignment. The one not conserved is the axial ligand to the (B)Chl in the Chl_Z/D_ position. However, this residue is found in a region of very poor sequence homology where a large loop insertion is present between TMH7 and 8 on the donor side, making the sequence alignment here unreliable. Since this axial ligand and (B)Chl position is conserved in all other known RCs, an interesting research direction will be to determine whether the CabRC also maintains the Chl_Z/D_ position. 23 of the 24 antenna BChls per PshA in the HbRC structure exhibit obvious axial ligands. The CabRC does not conserve any of the axial ligands that coordinate antenna BChls with the HbRC that are not conserved with the GsbRC, suggesting that the antenna number and arrangement of the CabRC is highly similar to the GsbRC. Thus, it is also likely that the peripheral lipid/carotenoid-containing region (Figs. 5, 6) is also conserved in the CabRC as it is the GsbRC and PSII.

In the CfxRC1 sequence, 9 of the 11 obvious axial ligands to BChls in each PscA of the GsbRC structure are conserved. Of the two that are not, one maintains a negatively charged sidechain (Glu in GsbRC, Asp in CfxRC1) and so likely maintains this BChl antenna site, and the other is an Asn instead of His in the GsbRC, which may also be involved in axial ligation. Both of these (B)Chl positions are conserved in the GsbRC, HbRC, PSI, and PSII, making it likely that they, too, are conserved in the CfxRC1 structure despite minor sequence differences. As was also the case with the CabRC, none of the HbRC antenna axial ligands are conserved in the CfxRC1 unless they are also conserved with the GsbRC, again suggesting a similar antenna number and arrangement to the GsbRC structure. We note that for both the CabRC and CfxRC1 structures, unique antenna sites cannot be predicted by sequence homology.

#### Fenna–Matthews–Olson protein and carotenoids

Though the GsbRC structure has only a single FMO attached, up to two have been observed to be associated with the complex in vivo, one on each side of the homodimeric core (Oh-oka et al. 1995; Bína et al. 2016). Previously, the FMO trimer was thought to be oriented approximately parallel to the membrane (Wen et al. 2009). The cryo-EM structure suggests that the FMO trimer is tilted ~ 15° relative to the membrane plane. The hydrophobic thickness prediction suggests that the lowest monomer of the FMO trimer skims along the top of the membrane (Supplementary Fig. 15). It may be that in detergent-solubilized GsbRCs the FMO has more flexibility to access the would-be membrane region, making this structural observation biologically irrelevant. Another interaction absent from detergent-solubilized GsbRC is that of the chlorosome baseplate with the FMO, which may also impact the FMO orientation.

Due to the FMO’s tilt and position relative to the core antenna, each monomer of the FMO trimer exhibits different BChl–BChl distances with the core. The shortest distance between a BChl in an FMO monomer to a BChl in the core is 21.60 Å from BChl 3 in the FMO to BChl 808. This FMO monomer is also that which we describe above as skimming the membrane (Supplementary Fig. 15). The second closest monomer exhibits a similar distance, 22.31 Å, however, the nearest interaction is between BChl 4 in the FMO and BChl 810 in the core. The close distances of BChl 3 and 4 to the core are consistent with the determination that they are lower energy relative to other BChls found in FMO and are therefore the ones transferring the excitation to the core (Milder et al. 2010). Finally, the furthest FMO monomer is substantially further away, 33.67 Å. This arrangement suggests an asymmetric energy transfer mechanism from the trimeric FMO to the core. Though FMO monomers are thought to be weakly energetically coupled, the observed distances in the structure suggest that when connected to the RC core, excitation in the furthest FMO monomer may preferentially transfer energy to an adjacent monomer rather than directly to the core. The other two FMO monomers, however, are similar distances from the core, and therefore probably transfer energy similarly. We wish to reiterate that this assumes that the FMO trimer orientation relative to the GsbRC is the same in the cryo-EM structure as it is in vivo.

Both BChl 808 and 810 sites are conserved in the HbRC, PSI, and PSII, and in the GsbRC exhibit axial coordination via PscA-His209 and PscA-His282, respectively. These axial ligands in the GsbRC would be interesting mutagenesis targets for energy transfer studies because they would probably impact energy transfer from the FMO trimer. The CabRC conserves both of the axial His residues and the CfxRC1 conserves the His corresponding the axial ligand for BChl 808 but replaces His for Gln for BChl 810, likely making a water molecule the axial ligand as in PSI.

The hydrocarbon tail of F26 is positioned in between BChl 808 and BChl 810, ~ 4 Å from either. F26 carotenoids are known to be additionally associated with chlorosomes, and the position of the F26 bound to the core near the FMO interface in the GsbRC structure suggests its involvement in energy transfer or quenching. BChl 810 is close to BChl 805, which is < 4 Å from the cyclic end of the F39 carotenoid. F39 is a glycosylated carotenoid that is bound only to the GsbRC core (Takaichi and Oh-oka 1999) in a region that shows structural conservation with PSII as noted above. A GsbRC mutant lacking carotenoid glycosylation shows growth defects under high light and slower quenching of the core antenna BChl *a* fluorescence (Azai et al. 2020); therefore, the F39 in the GsbRC must be responsible for this mutant phenotype, quenching excitation energy of BChl 808 via BChl 805. Though green sulfur bacteria typically reside in anaerobic environments, it is clear that mechanisms are present for coping with at least low levels of oxygen. Namely, they express various antioxidant proteins (Li et al. 2009) and the FMOs contain redox-active Cys residues for energy quenching (Orf 2016; Higgins 2021). The role of F39 in energy quenching is an important addition to our understanding of how green sulfur bacteria tolerate oxygen, but the surprising conservation of structural features in the F39 region between the GsbRC and PSII suggest that the incorporation of energy quenching mechanisms has been a powerful force in RC evolution, possibly from early on.

## Open questions and outlook

We have described many ways in which the GsbRC has provided fundamental insight into our understanding of RC structure, function, and evolution. Important comparisons were made regarding RC donor surfaces, ET chain cofactor identities and coordination, acceptor binding characteristics, and antenna pigment arrangement. Roles of the conserved donor-side Ca^2+^ ion, lipid involvement in the coordination of A_0_, and photoprotective carotenoids in the GsbRC are all important avenues for future research. Further insights into these topics may be provided by the solving of a more complete GsbRC structure with the PscC subunits bound. We have also provided insight into two RC1s that are distantly related to the GsbRC by comparing them to RCs that have been more extensively studied. The CabRC probably also coordinates the Ca^2+^ ion observed in the GsbRC and HbRC, but otherwise their donor surfaces are very different. Though electron donors and acceptors to the CabRC and CfxRC1 are challenging to predict, homology modeling confidently suggests that the antenna arrangement of these RCs is quite similar to that observed in the GsbRC. Importantly, this antenna arrangement appears more similar to the CP43 and CP47 subunits of PSII in both the number of (B)Chl sites and the positions, especially near a region that uniquely binds lipids and a carotenoid, the latter of which is important for energy quenching in the GsbRC core. The surprising similarities in structural characteristics between the GsbRC and PSII support proposals that place the development of water oxidation closer to the emergence of RCs than previously anticipated. A better understanding of structure, function, and diversity of homodimeric RC1s might also provide unexpected insight into the emergence of oxygenic photosynthesis that has shaped life as we know it.

## Supplementary Information

Below is the link to the electronic supplementary material.

Supplementary file1 (DOCX 2599 kb)
