# Supplementary material for: Recent advances in the structural diversity of reaction centers
Source: Photosynth Res. 2021 Jun 26;149(3):329–43. doi: 10.1007/s11120-021-00857-9 (PMC8452559; doi:10.1007/s11120-021-00857-9)
Supplement: Supplementary file 1 — Supplementary file1 (DOCX 2599 kb) [file 11120_2021_857_MOESM1_ESM.docx]

**Supplementary Information for**

Recent advances in the structural diversity of reaction centers

Christopher J. Gisriel^1,^*, Chihiro Azai^2,3^, Tanai Cardona^4^

^1^Department of Chemistry, Yale University, New Haven, CT, 06520, USA.

^2^College of Life Sciences, Ritsumeikan University, Kusatsu, 525-8577, Japan.

^3^Graduate School of Life Sciences, Ritsumeikan University, Kusatsu, 525-8577, Japan.

^4^Department of Life Sciences, Imperial College, London, UK.

*To whom correspondence should be addressed: [christopher.gisriel@yale.edu](file:///C:\Users\gisri\Desktop\GsbRC_paper\christopher.gisriel@yale.edu)

**Fig. S1.** Nomenclature for antenna domain, ET domain, and ET cofactors.

**Fig. S2.** Phylogenetic analysis of type I RC core polypeptide sequences.

**Fig. S3.** Electron donor and RC donor-side electrostatics surfaces from PSI, the GsbRC and the HbRC structures.

**Fig. S4.** Comparison of P-helices in type I RCs.

**Fig. S5.** Structural details of the Ca^2+^-binding site.

**Fig. S6.** Possible H-bond donors near 13^2^ methoxycarbonyl of P_700_.

**Fig. S7.** Experimental maps associated with the GsbRC and HbRC structures in the region of the 13^2^ methoxycarbonyl substituent of P and a nearby conserved Thr residue.

**Fig. S8.** Coordination of the Acc (B)Chl.

**Fig. S9.** Tetrapyrrole ring substituent coordination of the A_0_ (B)Chl.

**Fig. S10.** H-bonding network near A_0_ in the HbRC and corresponding region of the GsbRC.

**Fig. S11.** Example of density corresponding to a conserved water molecule in a cryo-EM map where waters are not assigned due to resolution.

**Fig. S12.** Possible Fd binding in the GsbRC.

**Fig. S13.** BChl site comparison of the GsbRC and HbRC.

**Fig. S14.** Sequence alignment of the GsbRC PscA, HbRC PshA, CabRC PscA, and CfxRC1 PscA.

**Fig. S15.** Possible interaction of the FMO protein with the lipid bilayer.

**Table S1.** Predicted hydrophobic thickness of representative RCs using the Positioning of Proteins in Membranes server.

**Table S2.** GsbRC antenna BChl site comparison with positions in the HbRC, PSI, and PSII.

**Supplementary Figures**


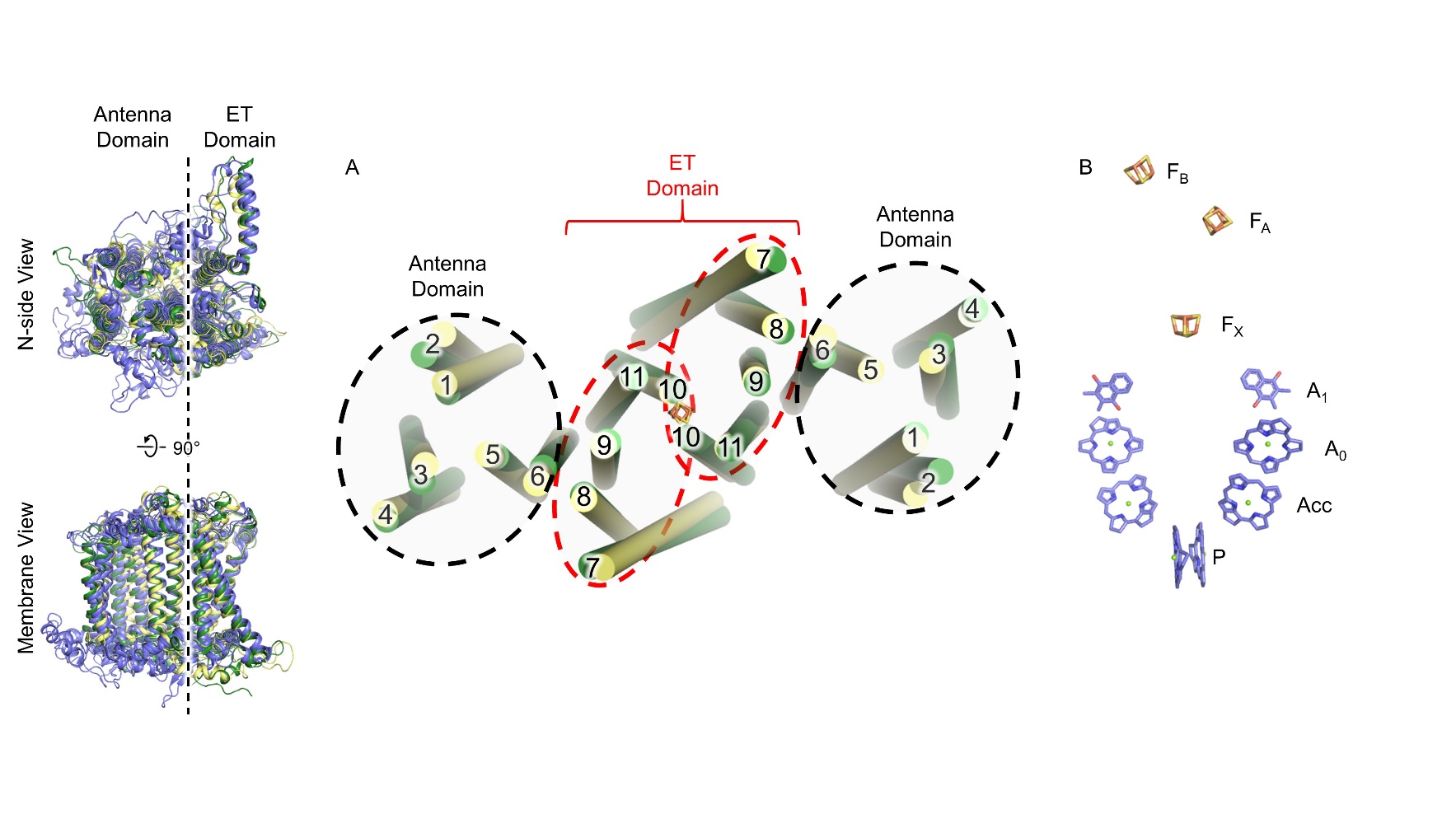


**Fig. S1.** Nomenclature for antenna domain, ET domain, and ET cofactors. **A** shows the donor-side view of the TMHs from the core subunits of the HbRC (yellow, PDB 5V8K) and GsbRC (green, PDB 6M32) structures. TMHs are labeled from N- to C-terminus and the [4Fe-4S] cluster termed F_X_ from each RC is shown for orientation. **B** shows the ET cofactors from PSI and the corresponding site nomenclature established previously (Jordan et al. 2001), used in the GsbRC (Chen et al. 2020) and HbRC (Gisriel et al. 2017) structure publications, and used herein. Hydrocarbon tails and Chl substituents are omitted for clarity.


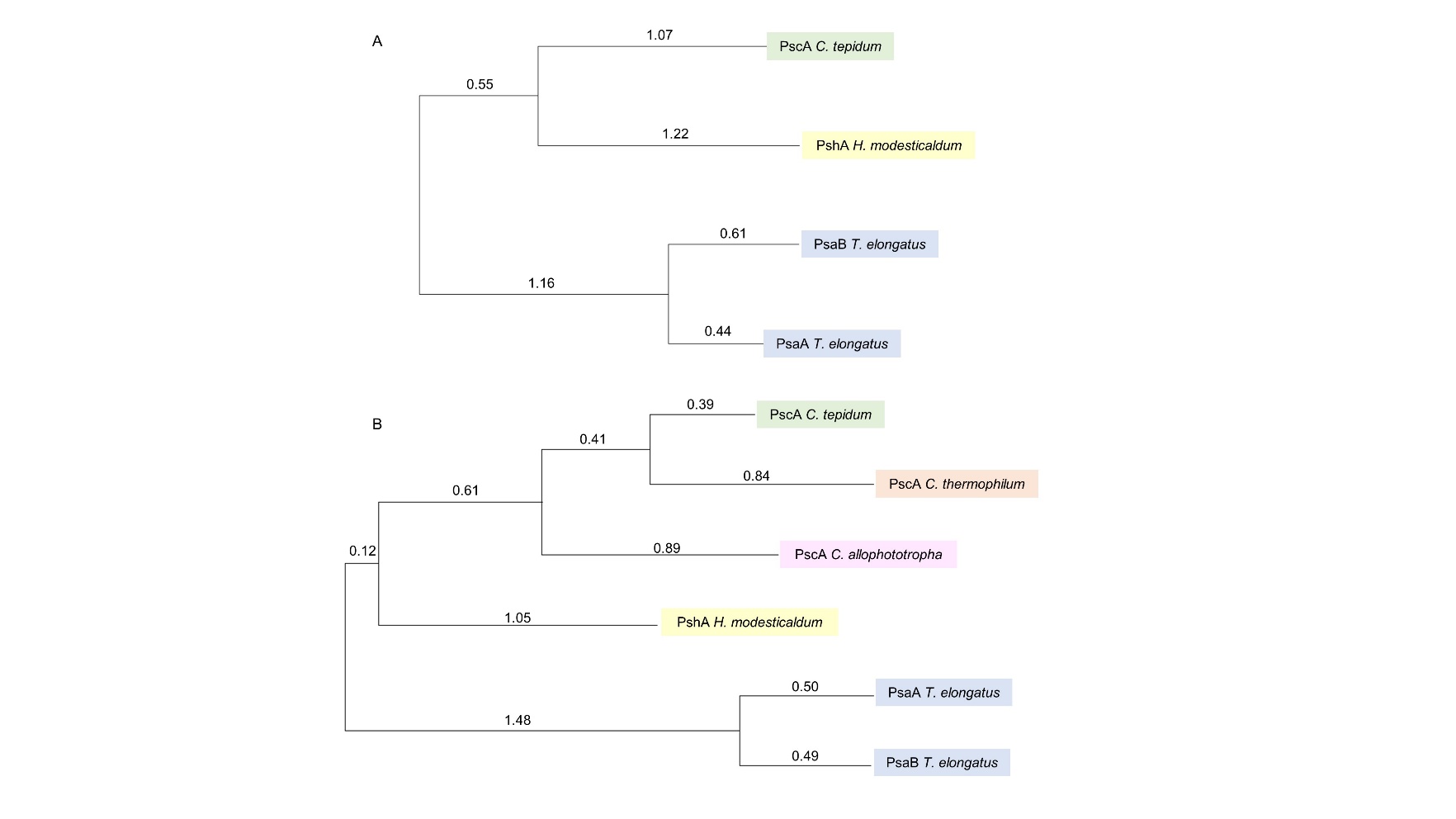


**Fig. S2.** Phylogenetic analysis of type I RC core polypeptide sequences.
For **A**, structure-based sequence alignments were created using the PROMALS3D server (Pei et al. 2008) and for **B**, sequence alignments were created using Clustal Omega (Sievers et al. 2011). The evolutionary history was inferred by using the Maximum Likelihood method based on the Le Gascuel model (Le and Gascuel 2008). The tree with the highest log likelihood (-6928.45 for **A** and -10618.99 for **B**) is shown. Initial tree(s) for the heuristic search were obtained automatically by applying Neighbor-Join and BioNJ algorithms to a matrix of pairwise distances estimated using a JTT model, and then selecting the topology with superior log likelihood value. A discrete Gamma distribution was used to model evolutionary rate differences among sites (3 categories [+*G*, parameter = 5.4756 for **A** and +*G*, parameter = 4.2418 for **B**]). The tree is drawn to scale, with branch lengths measured in the number of substitutions per site (value above the branches). The analysis involved 4 amino acid sequences for **A** and 6 amino acid sequences for **B**. There was a total of 934 and 1095 positions in the final dataset for **A** and **B**, respectively. Evolutionary analyses were conducted in MEGA7 (Kumar et al. 2016).


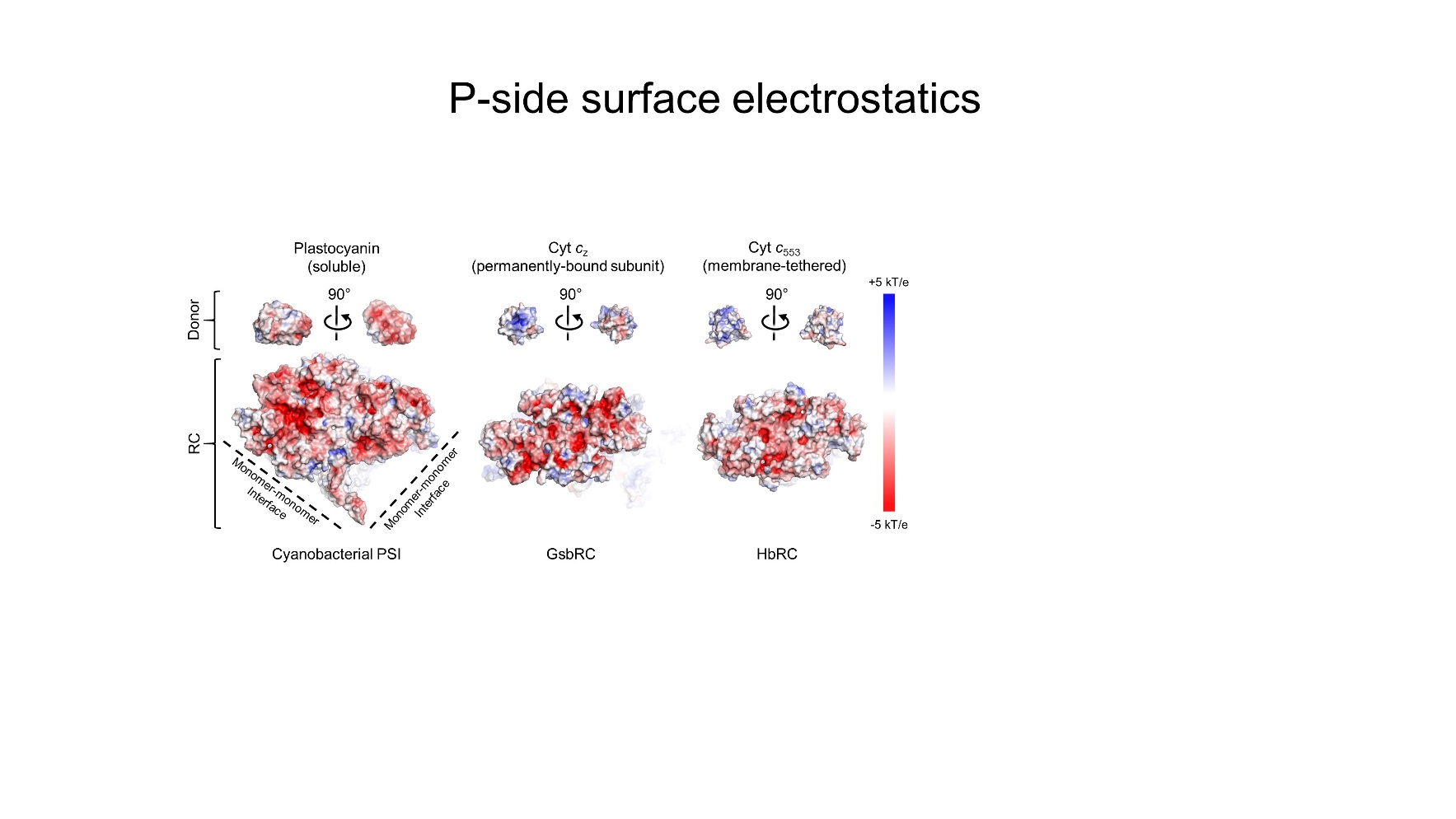


**Fig. S3.** Electron donor and RC donor-side electrostatics surfaces from PSI, the GsbRC and the HbRC structures. The electrostatic potential surface is shown for the RC’s donor-side and two views of each electron donor. Left shows cyanobacterial PSI (PDB 1JB0) and plastocyanin (PDB 1BXU), center shows the GsbRC (PDB 6M32) and cyt *c*_z_ (PscC) lacking the transmembrane domain (PDB 3A9F), and right shows the HbRC (PDB 5V8K) and a homology model of cyt *c*_553_ (SwissModel (Guex et al. 2009) from template 2C1B).


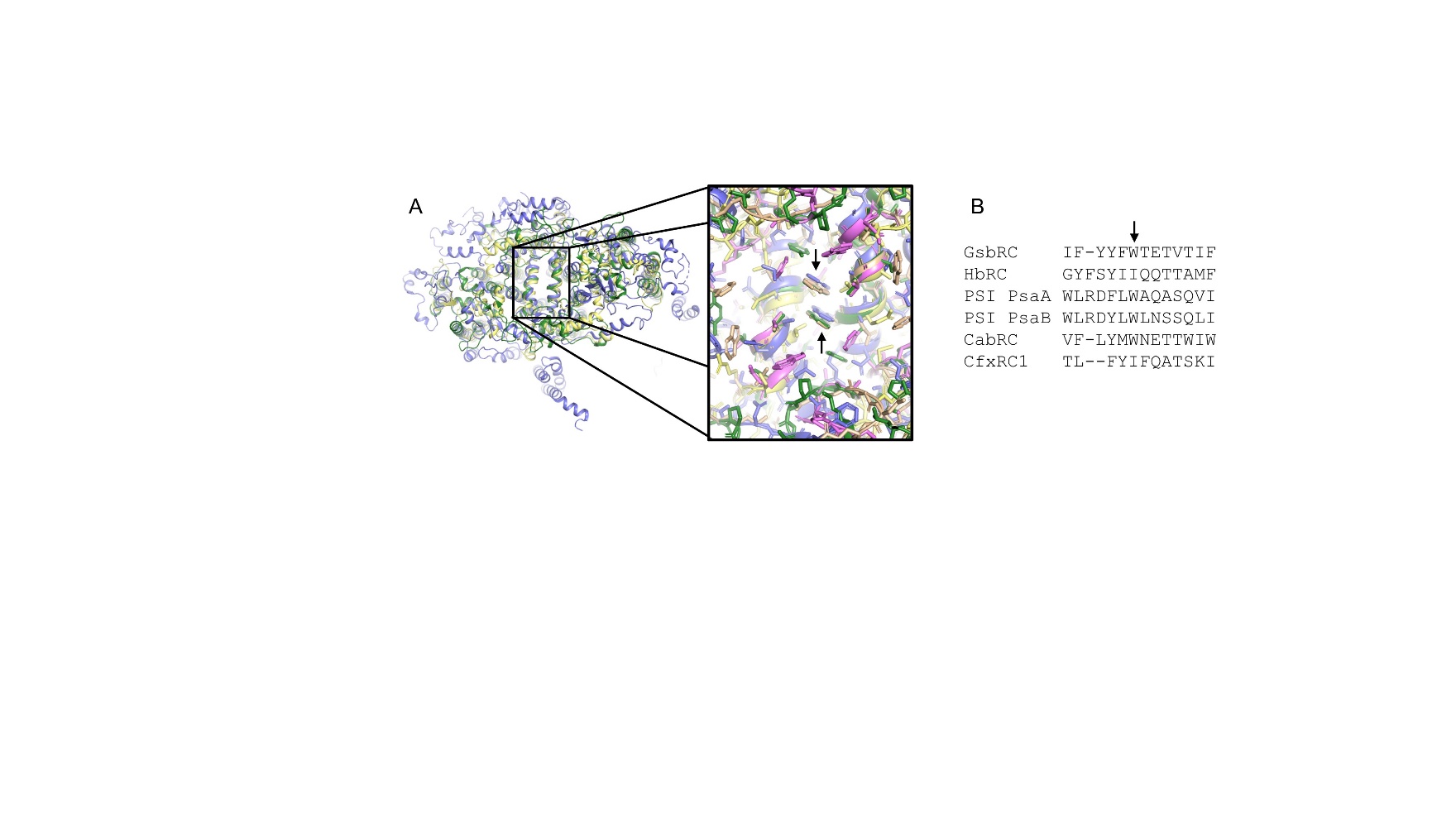


**Fig. S4.** Comparison of P-helices in type I RCs. **A** Superposition of experimentally-derived structures is shown (GsbRC = green, HbRC = yellow, PSI = blue). The magnification focuses on the P-helices and additionally includes homology models of the CabRC and CfxRC1 (CabRC = wheat, CfxRC1 = magenta). **B** Structure-based sequence alignment (Pei et al. 2008) of only the P-helix region. The black arrows designate the Trp residue shown by Sommer et al. to be important for electron transfer from plastocyanin or cytochrome *c*_6_ to P_700_^+^ (Sommer et al. 2004; Hippler and Drepper 2006).


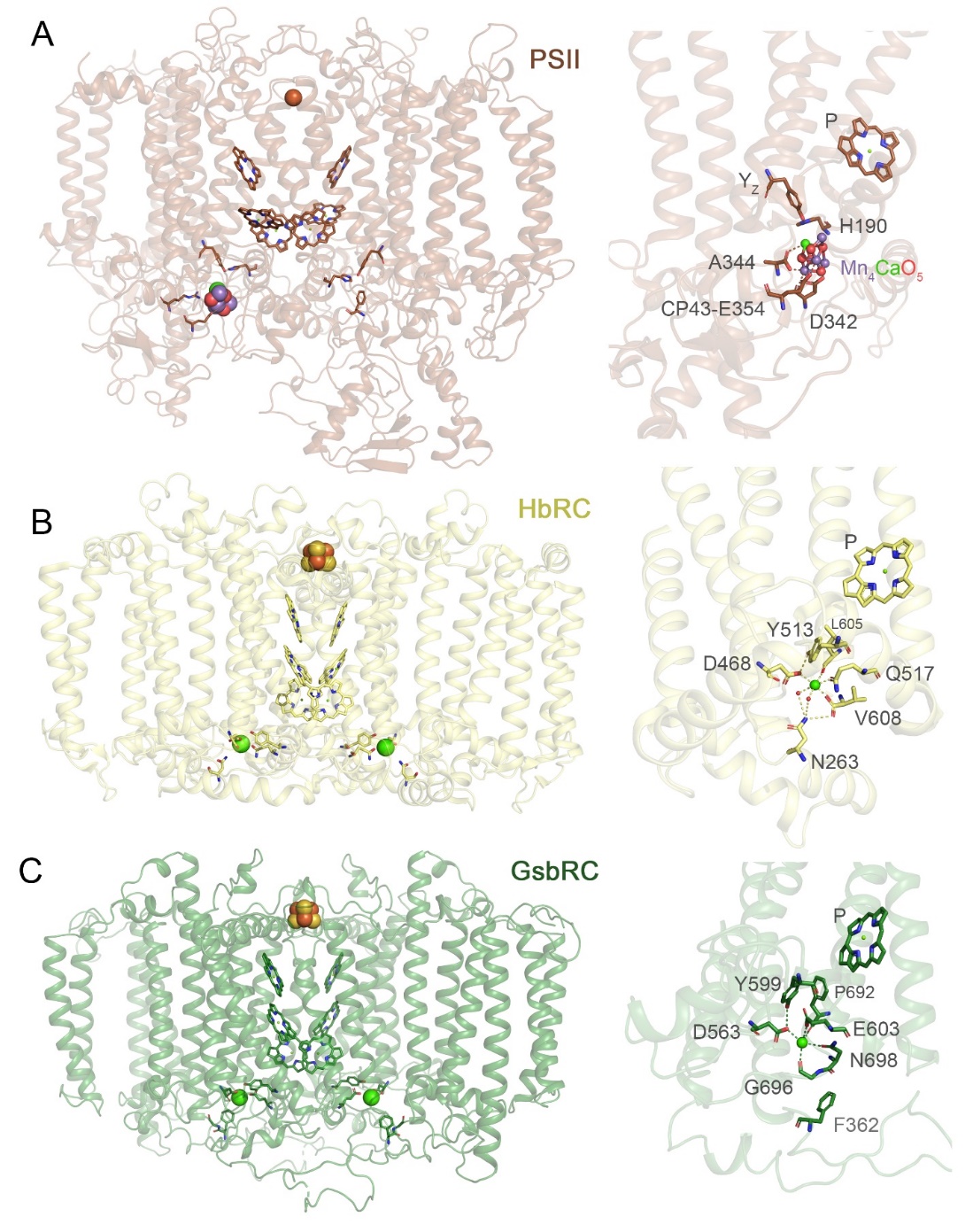


**Fig. S5.** Structural details of the Ca^2+^-binding site. **A** shows PSII, **B** shows the HbRC, and **C** shows the GsbRC in brown, yellow, and green, respectively. The left column shows a transmembrane view of the complexes, highlighting the position of the Mn_4_CaO_5_ cluster of PSII relative to the Ca^2+^ site in the RC1 complexes. The right column shows a detailed view of the region near the Mn_4_CaO_5_ cluster or Ca^2+^ site. The Ca^2+^ bound by the HbRC is coordinated by PshA-Asp468, Tyr513, Gln517, the carbonyl oxygen of PshA-Leu605 and the carboxylic C-terminus of PshA-Val608. The Ca^2+^ site has a slightly distorted octahedral coordination, with the fifth and six ligand positions occupied by two water molecules, both of which are within H-bonding distance of PshA-Asn263, located in the extrinsic loop between the 5^th^ and 6^th^ TMH of the antenna domain. In the GsbRC the Ca^2+^ is coordinated by residues PscA-Asp563, Tyr599, Glu603, and Phe692 at homologous positions. While the HbRC C-terminus ends sharply at the 11^th^ TMH, providing coordination to the Ca^2+^, the GsbRC PscA has a 36-residue C-terminal extension that folds around the Ca^2+^ site. At the end of the 11^th^ TMH, PscA-Asn698 and the carbonyl oxygen of PscA-Gly696 provide additional coordination, the latter occupying a position equivalent to one of the coordinating waters in the HbRC. In PSII, the Mn_4_CaO_5_ cluster is coordinated by CP43-Glu354 from the antenna’s extrinsic domain located between the 5^th^ and 6^th^ TMH of CP43, a structural arrangement that is mirrored in CP47. In the HbRC, the Ca^2+^ site is linked to the antenna domain via H-bonded water molecules. In contrast, in the GsbRC the antenna extrinsic domain does not directly interact with the site. Instead, PscC-Phe362 is in van der Waals contact with PscC-Gly696.


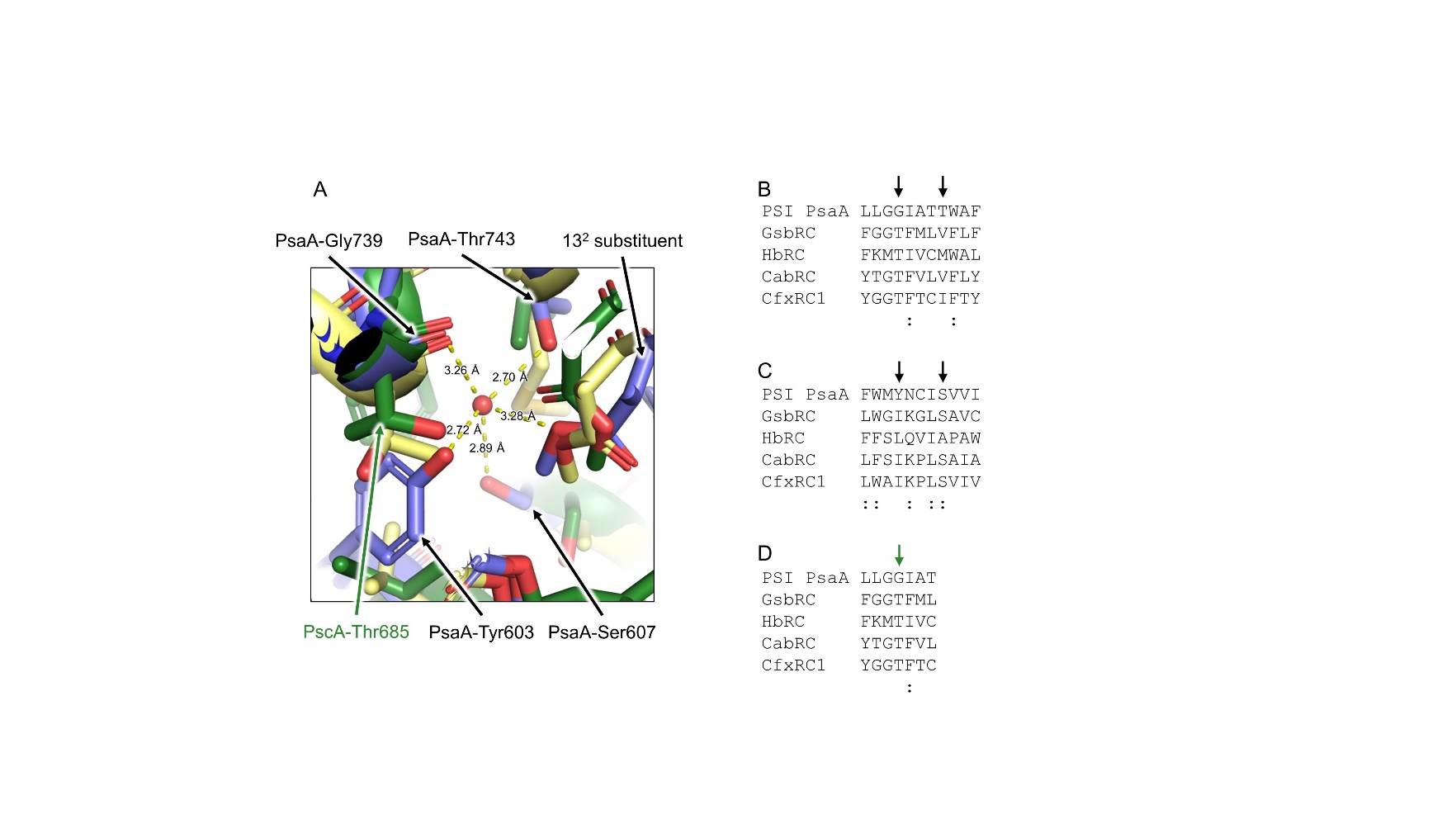


**Fig. S6.** Possible H-bond donors near the 13^2^ methoxycarbonyl moiety of P. **A** Superposition of GsbRC (green), PSI (blue, Chl *a*′ side), and HbRC (yellow) structures in the region of the 13^2^ methoxycarbonyl substituent. Note that the water molecule shown (red sphere) corresponds only to the PSI structure. In PSI, Jordan et al. suggested that a H-bonding network involving the 13^2^ methoxycarbonyl substituent of Chl *a*′, the water molecule, and the four residues labeled in black font and arrows is present (Jordan et al. 2001). In the HbRC, no H-bonding is observed near the 13^2^ methoxycarbonyl substituent on P_800_. The resolution of the GsbRC structure is insufficient to resolve water molecules or the orientation of the PscA-Thr685 (green font and arrow) residue that could be involved in H-bonding to the BChl *a*′ 13^2^ methoxycarbonyl substituent. **B-D** show the corresponding sequence alignments of residues labeled in **A**.


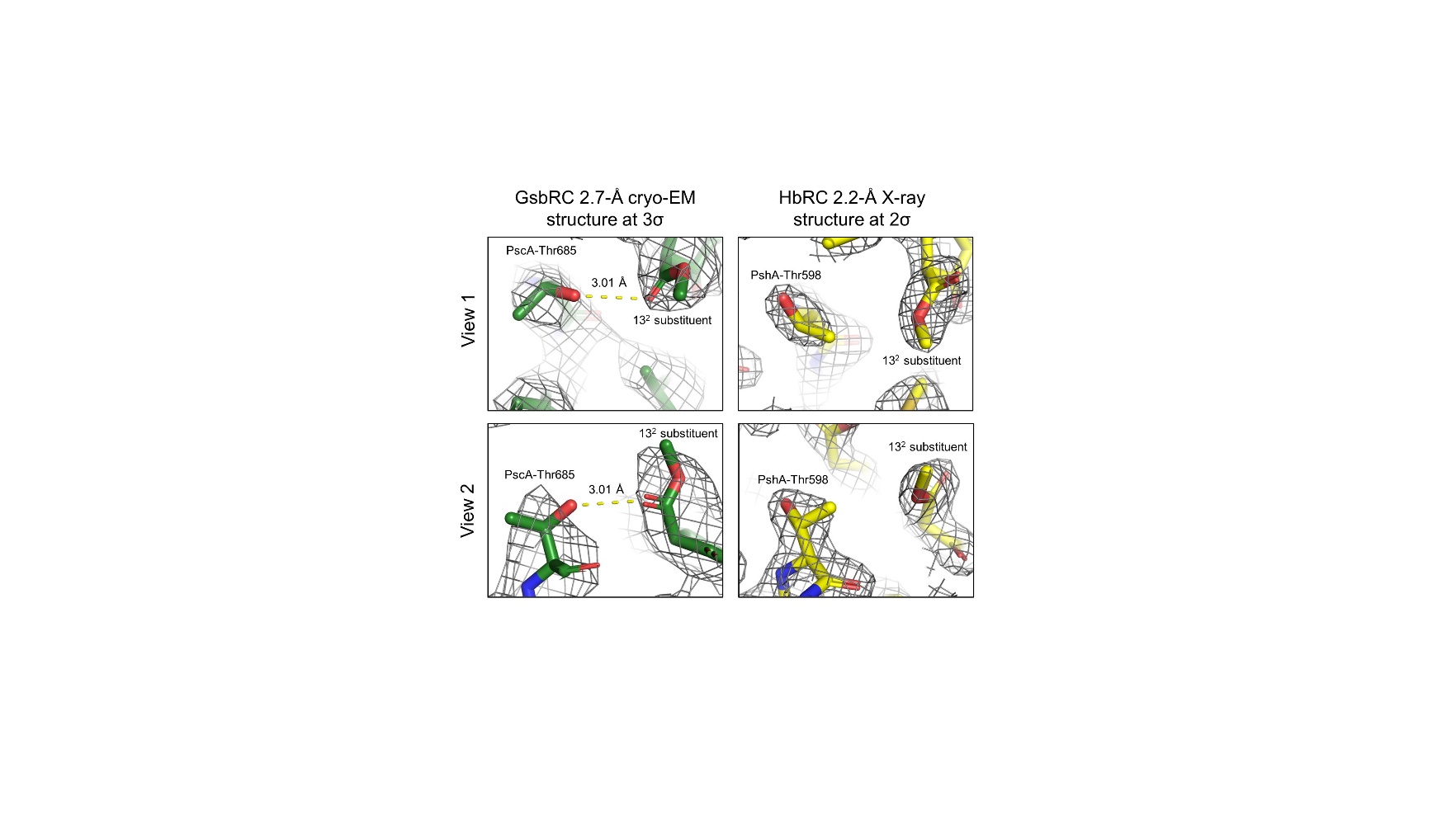


**Fig. S7.** Experimental maps associated with the GsbRC and HbRC structures in the region of the 13^2^ methoxycarbonyl substituent of P and a nearby conserved Thr residue. In the GsbRC (two left panels), the map resolution is too low to distinguish the orientation of PscA-Thr685 and the 13^2^ substituent, making their orientation in the model arbitrary. In the HbRC (two right panels), the map resolution is high enough to accurately model the positions of PshA-Thr598 and the 13^2^ substituent.


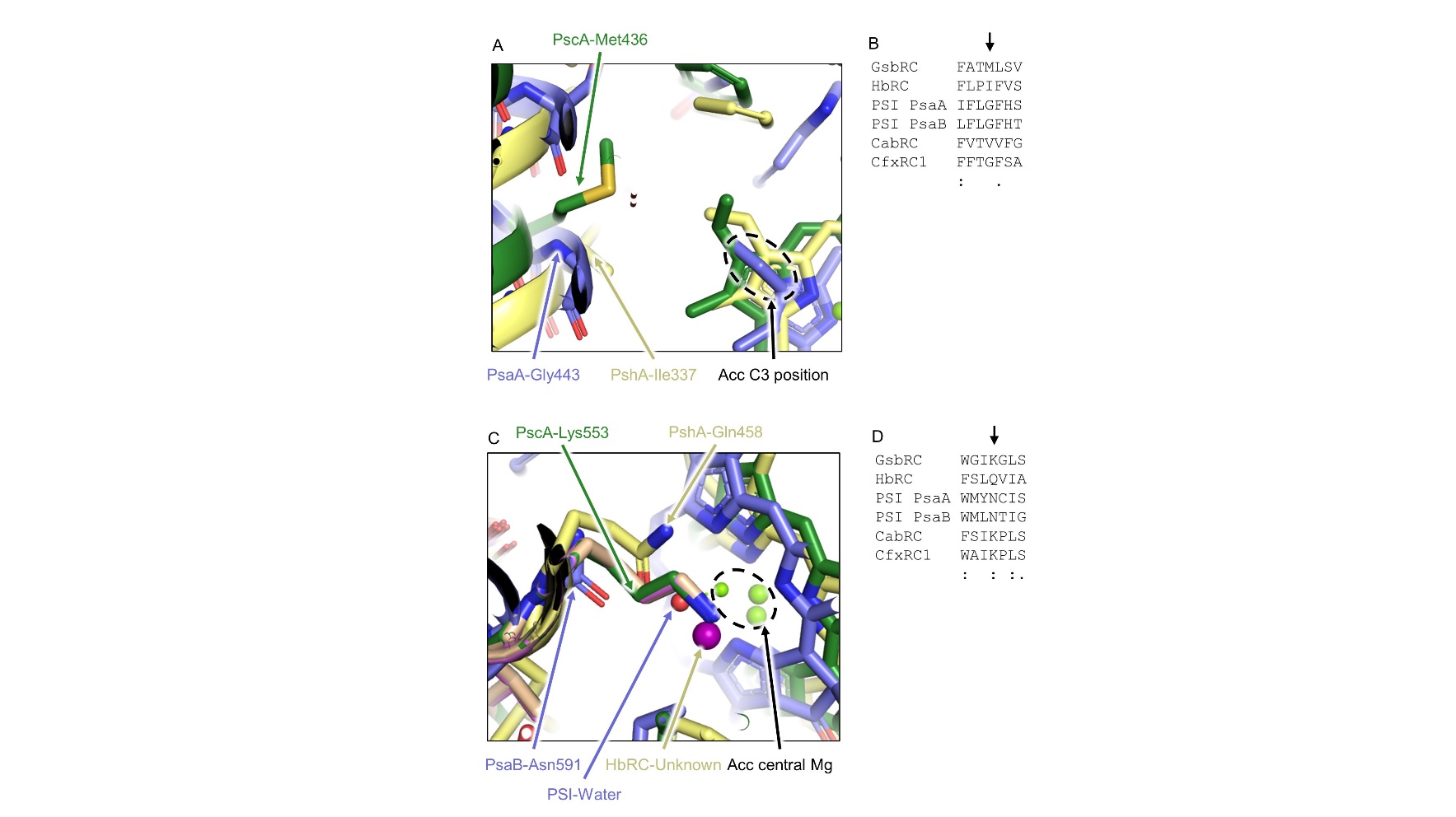


**Fig. S8.** Coordination of the Acc (B)Chl. **A** and **C** show the superposition of structures near the Acc position for the C3 substituent and axial ligand, respectively. For **A**, PscA-Met436 and analogous residues are labeled and **B** shows the sequence alignment corresponding to relevant residues in **A**, additionally including the sequences from PsaB, PscA from the CabRC, and PscA from the CfxRC1. For **C**, participants in axial ligation are shown. Note that the water molecule (red sphere) is part of the PSI structure and the purple sphere represents a putative ion in the HbRC structure. **D** shows the sequence alignment corresponding to relevant residues in **C**, additionally including PsaA. In **A** and **C**, the GsbRC is shown in green, HbRC in yellow, and PSI in blue. **C** also shows the homology models for the CabRC in wheat and the CfxRC1 in magenta.


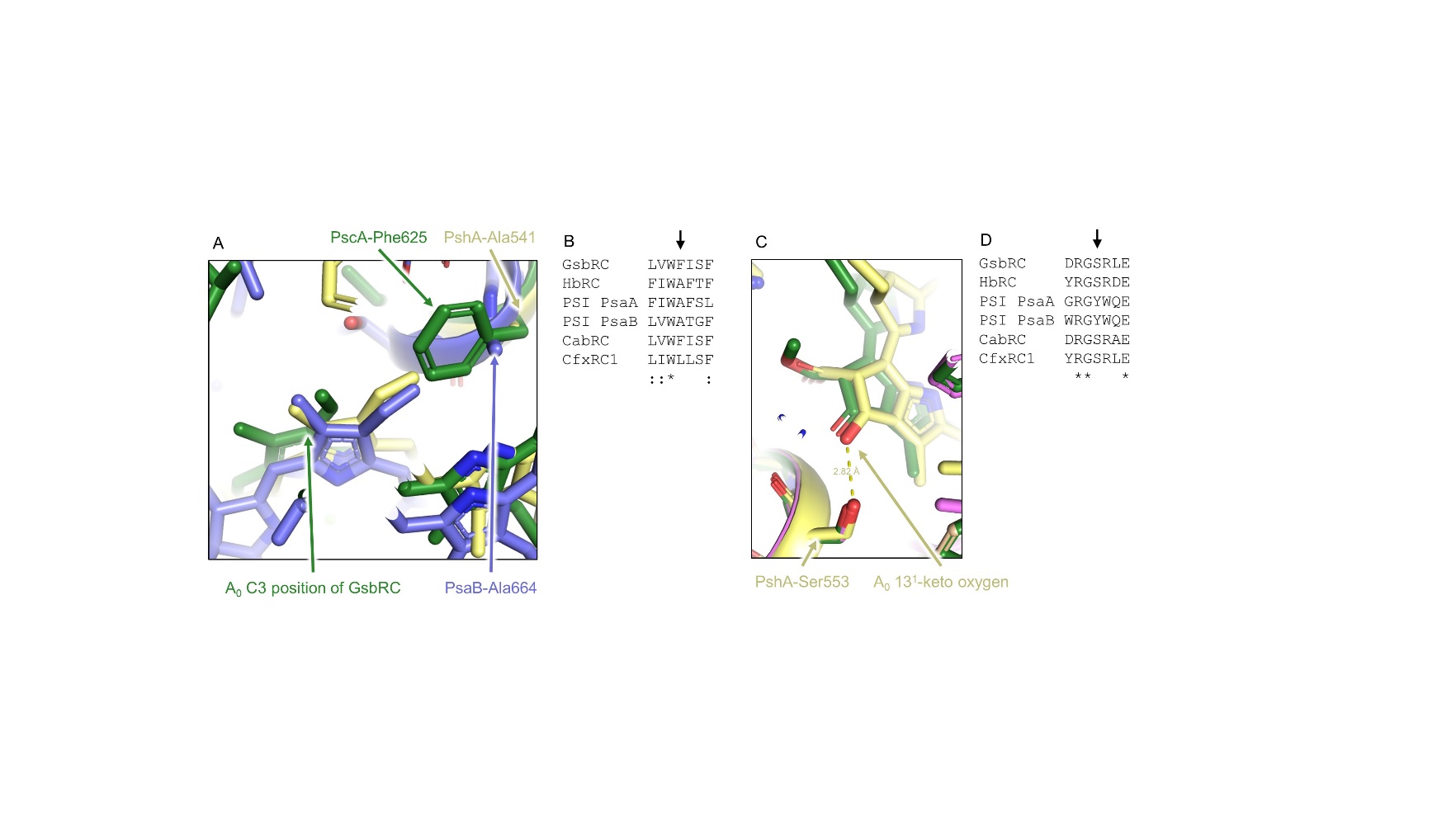


**Fig. S9.** Tetrapyrrole ring substituent coordination of the A_0_ (B)Chl. **A** and **C** show the superposition of structures near the A_0_ position for the C3 substituent and 13^1^-keto oxygen, respectively. For **A**, PscA-Phe625 and analogous residues are labeled and **B** shows the corresponding sequence alignment additionally including the sequences from PsaA, PscA from the CabRC, and PscA from the CfxRC1. **C** shows the conserved H-bond donation to the 13^1^-keto oxygen of A_0_ and **D** shows the corresponding sequence alignment. In **A** and **C**, the GsbRC is colored green, HbRC is colored yellow, PSI is colored blue, and homology models for the CabRC and CfxRC1 are colored in wheat and magenta, respectively.


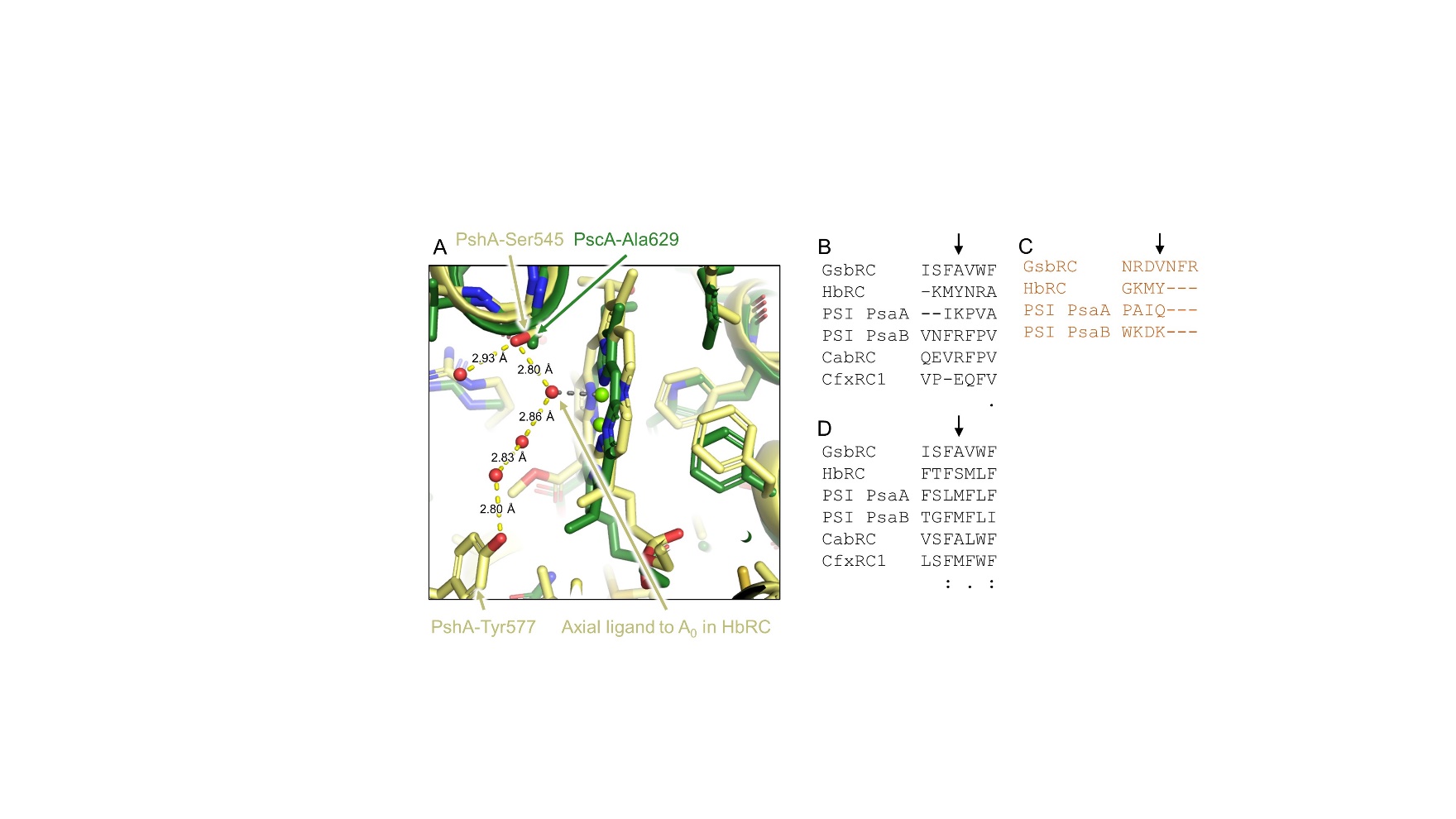


**Fig. S10.** H-bonding network near A_0_ in the HbRC and corresponding region of the GsbRC. **A** The structural superposition of the GsbRC (green) and HbRC (yellow). Water molecules (red spheres) are only present in the HbRC structure. The H-bonding network involved in providing the axial ligand to A_0_ in the HbRC is shown. Neither of the residues involved in the water network in the HbRC are conserved in the GsbRC, suggesting against similar axial coordination. **B** The sequence alignment near PshA-Tyr557 showing no homology. **C** The structure-based sequence alignment (orange font) also showing no sequence conservation for PshA-Tyr557. **D** The sequence alignment near PshA-Ser545 showing no sequence conservation.


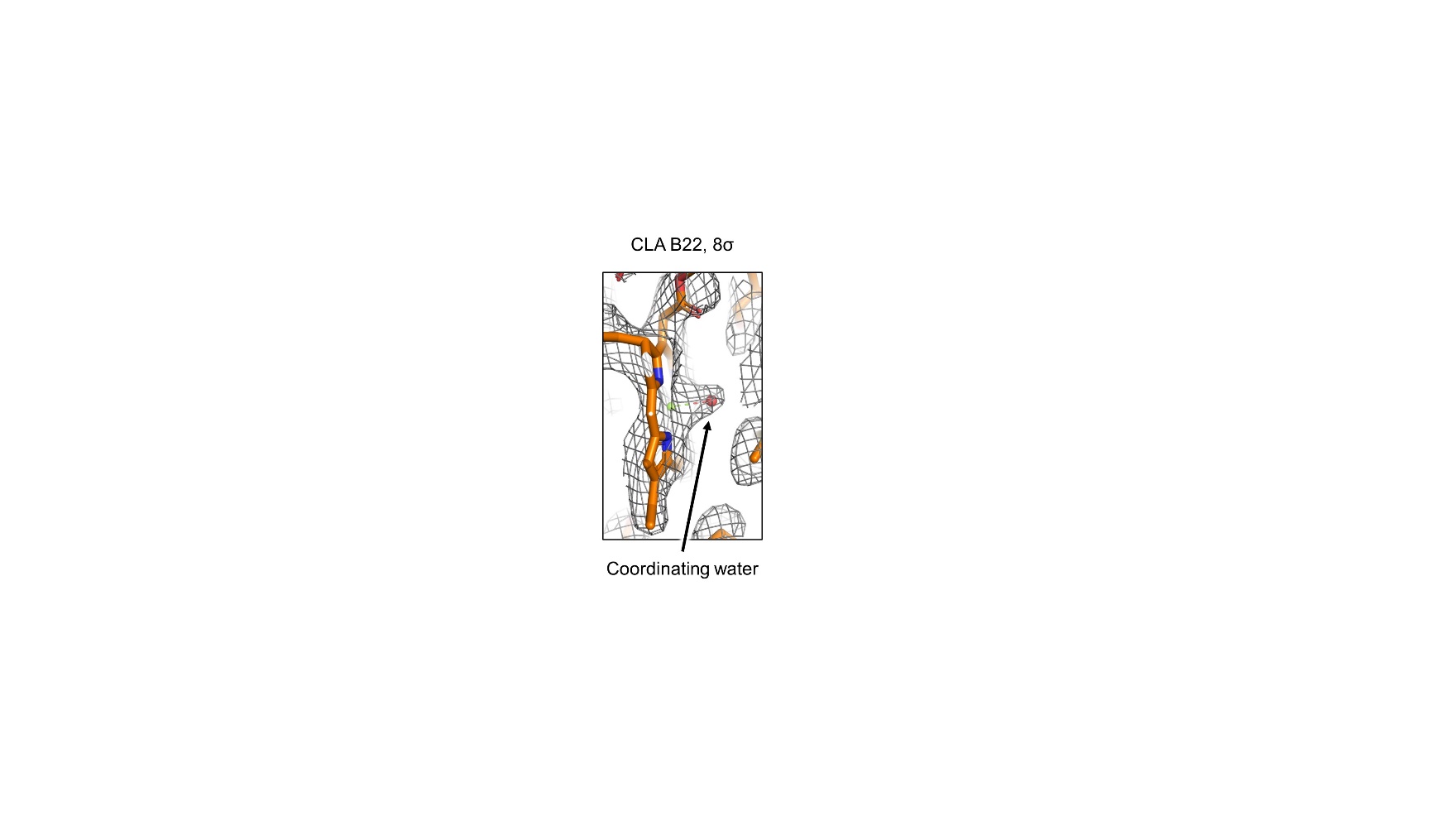


**Fig. S11.** Example of density corresponding to a conserved water molecule in a cryo-EM map where waters are not assigned due to resolution. Chl B22 is shown from the *T. elongatus* PSI cryo-EM structure (PDB 6TRA, residue number 826 in the PDB file) where axial coordination by a water molecule is clearly visible at ~2.85-Å resolution (Kölsch et al. 2020), which is lower than that of the GsbRC cryo-EM structure. This interaction is confirmed by the X-ray structure of PSI from the same organism (Jordan et al. 2001).


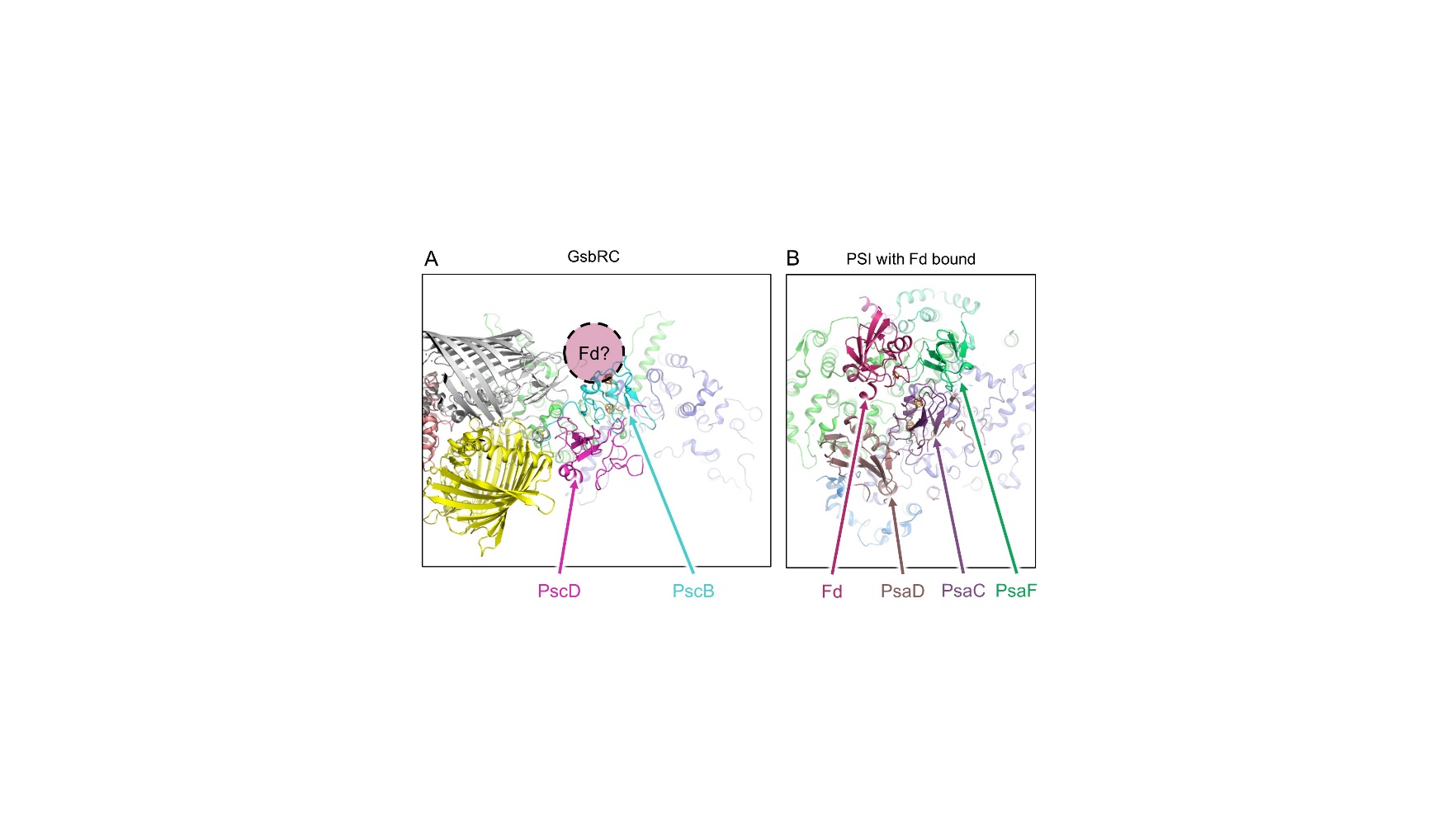


**Fig. S12.** Possible Fd binding in the GsbRC. In **A**, the position in the GsbRC analogous to the known Fd binding site in PSI is shown from an acceptor-side view. In **B**, the same view is shown but for PSI with Fd bound (PDB 5ZF0).


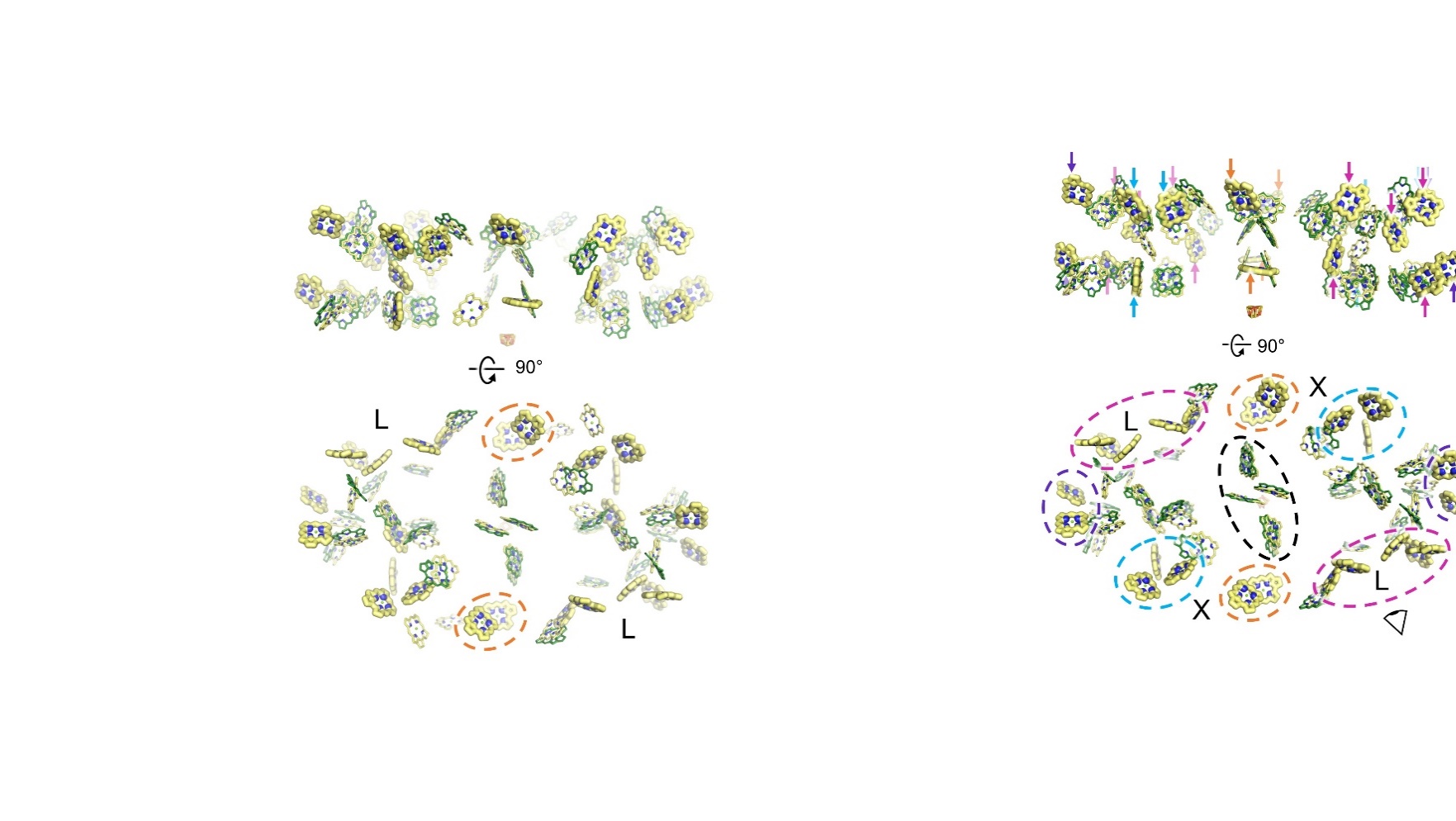


**Fig. S13.** BChl site comparison of the GsbRC (green) and HbRC (yellow). Those antenna BChls found in the HbRC but not in the GsbRC are shown with sticks of larger radii. The two BChls on each side of the ET chain circled in orange are among the closest to the ET chain in the HbRC but are not found in the GsbRC. The “L” label indicates the lipid and carotenoid-containing region conserved in the GsbRC and PSII but not the HbRC or PSI.

GsbRC PscA MAEQVKPAGVKPKGTVPPPKGNAPAPKANGAPGGASVIKEQDAAKMRRFLFQRTETRSTK 60

HbRC PshA ----------------------------------------MATAEMA------FNPRAQV 14

CabRC PscA -----------------------------------------------MASFSSYANGVKR 13

CfxRC1 PscA MSIQLRPA--------------------------APVINRPA-----LNKAAELHVRGYN 29

GsbRC PscA WYQIFDTEKLDDEQVVGGHLALLGVLGFIMGIYYISGIQVFPWGAPGFHDNWFY-LTIKP 119

HbRC PshA FEYFKDKVPATRGAVLKAHINHLGNVAAMVSFILVHHL---SWDPATQGVLWAPATMFYA 71

CabRC PscA WYQKLEL-PMPPERIFGAHMMLIGGLACLIGTYFFASMTMW-------NDGYVN-LTLRP 64

CfxRC1 PscA WYQLFAF-PRTEDQIFGGHLIVSGMIGVMVAFNIGSGITA--------PGNYFF-LSIEP 79

: :. .*: * :. ::. : : :

GsbRC PscA RMVSLGIDT-YSTK----------------------TADLEAAGARLLGWAAFHFLVGSV 156

HbRC PshA RLYQLGLDATALSPDA-------------------------------LFVARMHLLAA-- 98

CabRC PscA RLISLGIYDPYDTEQIQRVWLPLIGEFSTSKLPFFGQYPLTMTDFRLFGWGCFHIGLGLW 124

CfxRC1 PscA QLYNLGIDTKEK----------------------------------VLTYALIHIISGLG 105

:: .**: : . :*: .

GsbRC PscA LIFGGWRHWTHNLTNP--------------FTGRCGNFRDFRF----------LGKFGDV 192

HbRC PshA IILWGFGHVKSPAEEK-------------------------------------------- 114

CabRC PscA LVYAGAAHYYGARGGATIGEIFWLLPYVPGLKGLCQI-KWFTPEGPWYKVGLPWGSFANT 183

CfxRC1 PscA LILFGWWHYRNRIL---------------------------------------------- 119

:: * *

GsbRC PscA VFNGTSAKSYKEALGPHAVYMSLLFLGWGIVMWAILGFAPIPD----------------- 235

HbRC PshA ---FLEKVTMGKALVAQFHFFALIATLWGLHMAFYGIL-----GPSGKLEPTGL------ 160

CabRC PscA PW-PILRRTYADALSPHTIYIGLLFFIWGFVLWFVLDKPPVPLQPAQVMTPNGLMPLEQA 242

CfxRC1 PscA ---QAAALTYAKAWGGHLIYIAWWCFAWNLWSLLVMQRGPVLFGIG-------LTDAAQR 169

: .* : ::. *.:

GsbRC PscA -----------------FQTINSETFMSFVFAVIFFALGIYWWNNPPNAA-IHLN-DDMK 276

HbRC PshA --SFDMFGPITPATM------AG---NHVAFGAVFFLGGIFHYFAGFNTKRFAFFEKDWE 209

CabRC PscA PFPYGWFDPYLNQVMHPMNTINGETTMCFVWGVLFVALGAYWWYRPPRSINITHL-EDTK 301

CfxRC1 PscA Q-----------AKIIPL--MNFDGFLL-LLACALLIAGVVTYRKPPT-F-NRHL-EDPK 212

. :. * : .* :

GsbRC PscA AAFSVHLT--AIGYINIALGCIAFVAFQQPSFAPYYKELDKLVFYLYGEPFNRVSFNFVE 334

HbRC PshA AVLSVSCQILAFHFATVVFAMI---IWQHPQLGF--GFMREYAVSQYAGP---------- 254

CabRC PscA AVFHVHLT--AIGYVSFALAIVGFLALRNH---PSYLMLNDMNVIIYGK----------- 345

CfxRC1 PscA AQLHVNLV--IFGWTGMLYSAFAWFTFPRGE----------------------------- 241

* : * : : . . . .

GsbRC PscA QGGKVISGAKEFADFPAYAILPKSGEAFGMARVVTNLIVFNHIICGVLYVFAGVYHGGQY 394

HbRC PshA ----------------ELKMI----AQSNPGL-LVKQAILGHLVMGIMFWIGGVFHGAHF 293

CabRC PscA -------------------------KIVNPGRMIHNMITFNHVQVGLLYVAAGVFHGGQY 380

CfxRC1 PscA -----------------------SAALGQWSDVTLNLFVWNHVNCCALYIFGGVFHGAQY 278

. : .*: :: .**:**.::

GsbRC PscA LLKIQLNGMY--NQIKSIWITKGRDQEVQVKILGTVMALCFATMLSVYAVIVWNTICELN 452

HbRC PshA MLRVLNDPKLAEEMKDFKFIKRCYDHEFQKKFLALIMFGAFLPIFVSYGIATHNTIADIH 353

CabRC PscA LHGLNISGAY--KQARSKFITWFQNPDLQTKIVGTTMFVSFVTVVFGYGMICWNTGAELD 438

CfxRC1 PscA LWDVRDDDTK-NPWVISKWIRWFDKEDRQVDISVICFFFAFFTGFSAWGVMGINSLVDFK 337

: : . :* . : * .: : .* . :.: *: ::.

GsbRC PscA IFGTNI------------------------------------------------------ 458

HbRC PshA AASKTGLFAHMTY----------------------------------------------- 366

CabRC PscA LNFGIYQFRSFRAIQMDGEAGNIGYRVFRPKNPWDPTAGGDWVKNPDGTAKLVKARNLQV 498

CfxRC1 PscA IFPP-------------------------------------------------------- 341

GsbRC PscA ---------------TMSF----------YWLKP----------------L-PIF----- 471

HbRC PshA ----------------------------INIGTP-------------------LH----- 374

CabRC PscA GDRILNEELGIGSSPTYSFTTIEEINYKPEWGQPKLYAVQWGSWTHFLRKVNPLFWVDKG 558

CfxRC1 PscA -------------------------EWYIDWLRPYILETVWPGILHV------------- 363

*

GsbRC PscA QWM--------------------------------------------------------- 474

HbRC PshA ------------------------------------------------------------ 374

CabRC PscA IWYLQNQKTFEATRKADEAYLAAHLKAVSLLNQIDDAQTEEAKQKAQAELDKFRPELEKA 618

CfxRC1 PscA -W---------------------------------------------------------- 364

GsbRC PscA -------------------------FADPSINDWVMAHVITAGSLFSLIALVRIAFFAHT 509

HbRC PshA ---------------------DAIFGSKGSISEFVAAHAIAGGLHFTMVPMWRMVFFSKV 413

CabRC PscA HANMLEWNERLASTPAVLYSNLRDQHRDGEINDAIFFWLMIGGWLFGFIPLLRIAFHNYQ 678

CfxRC1 PscA ------------------------PGYQTSVSDWVFLHALTSGFFFVMIPLSRAVFFTRR 400

. .:.: : : .* * :: : * .*.

GsbRC PscA SPLWDDLGLK-KNSYSFPCLGPVYGGTCGVSIQDQLWFAMLWGIKGLSAVCWYIDGAWIA 568

HbRC PshA SPWTTKVGMKAKRDGEFPCLGPAYGGTCSISLVDQFYLAIFFSLQVIAPAWFYIDGCWMG 473

CabRC PscA SPWYRDFEWR-KQSPDFPCIGPVKGGTCGVSIQDQLWFCILFSIKPLSAIAWYLDGGWIA 737

CfxRC1 PscA SPLFDAKGIT-KRSFDYPCLGPAYGGTCGFSIQDQIWLAMLWAIKPLSVIVWYMMGAYFG 459

** *.. .:**:**. ****..*: **:::.:::.:: :: :*: * ::.

GsbRC PscA SMMYGVPAADAK---------AWDSIAHLHHHYTSGIFYYFWTETVTIFS--SSHLSTIL 617

HbRC PshA SFVAVAAPYNDIYQAALATFNSHNPLHQLSPLTNMGYFSYIIQQTTAMFSRYDGHMIQAL 533

CabRC PscA TMMARGNEAYY---------------LTHNISHTGGVFLYMWNETTWIWT--DNHLTAML 780

CfxRC1 PscA SFQYAIDPKDP--------------AGPLSFPGRPGTLFYIFQATSKIWN--DGHLTMIL 503

:: * : *: * ::. ..*: *

GsbRC PscA MIGHLVWFISFAVWFEDRGSRLEGADIQTRTIRWLGKKFLNR-DVNFRFPVLTISDSKLA 676

HbRC PshA LGAHFIWAFTFSMLFQYRGSRDEGAMVLKWAHEQVGVGFAG----KMYNRALSLKEGKAI 589

CabRC PscA LLGHLIWFVSFALWFKDRGSRAEGGDIQSRWVRLMGKRLGIKTLQEVRFPVSNLATAKLW 840

CfxRC1 PscA YYGHLIWLLSFMFWFMYRGSRLEGAYILSRFLKR---HFNIN-LVP-EQFVTTLVGSRFL 558

.*::* .:* . * **** **. : . . : . .: .:

GsbRC PscA GTFLYFGGTFMLVFLFLANGFYQTNS-------PLPPPVSHAAVSGQQMLAQLVDTLMKM 729

HbRC PshA GCFLFFKMTIVCMWALAMV----------------------------------------- 608

CabRC PscA GTVFFYTGTFVLVFLYFADGFFQNR----------------------------------- 865

CfxRC1 PscA GTCFYYGGTFTCIFTYLAVGYSWRPNIGVPVLVPLPAP---------------------- 596

* ::: *: ::

GsbRC PscA IA 731

HbRC PshA -- 608

CabRC PscA -- 865

CfxRC1 PscA -- 596

**Fig. S14.** Sequence alignment of the GsbRC PscA, HbRC PshA, CabRC PscA, and CfxRC1 PscA. Residues involved in the axial ligation of antenna BChls are highlighted in green and residues involved in the axial ligation of ETC (B)Chls are highlighted in yellow. The alignment was performed with Clustal Omega (Sievers et al. 2011).


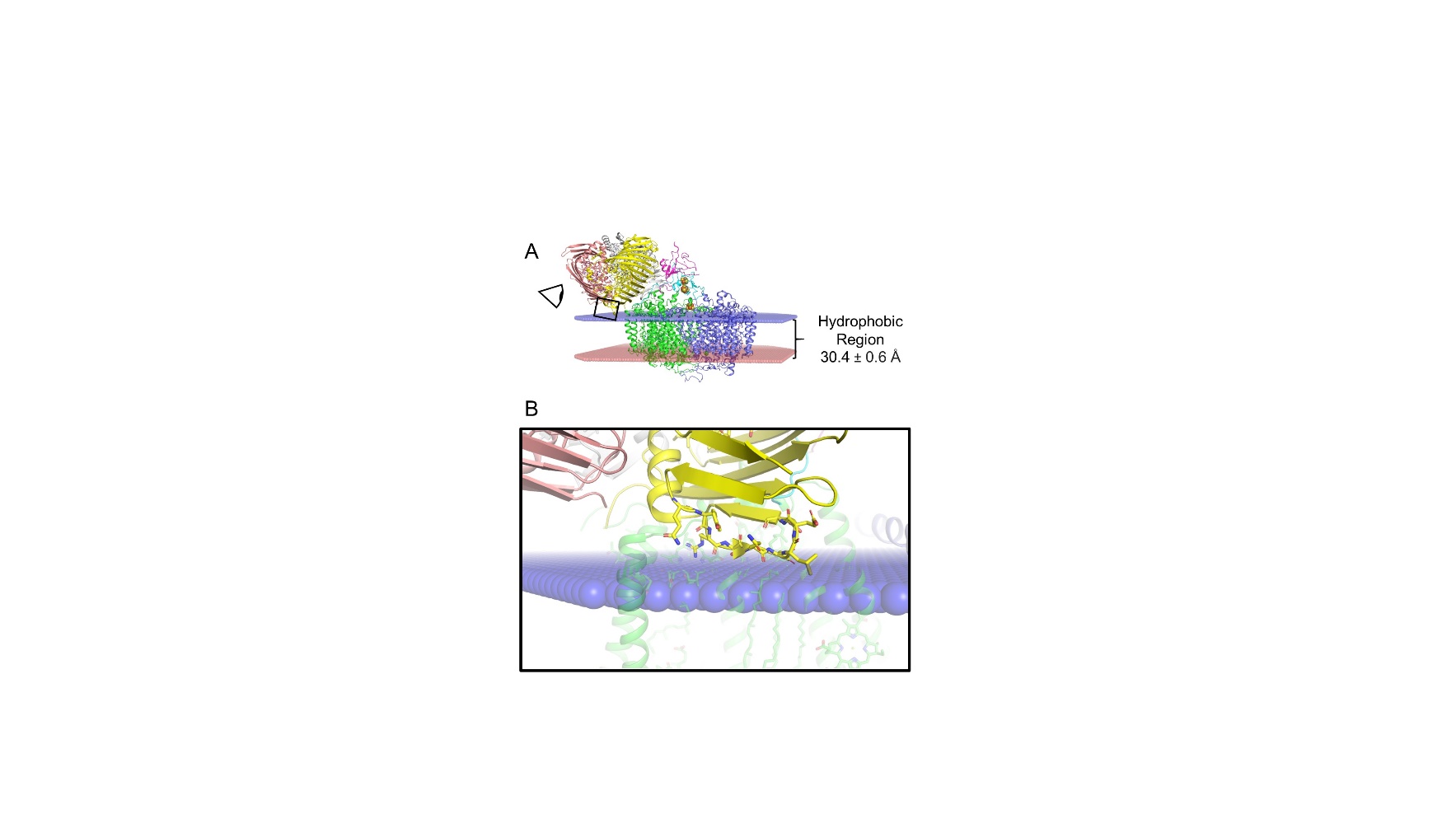


**Fig. S15.** Possible interaction of the FMO protein with the lipid bilayer. **A** Overall view of the GsbRC and its predicted hydrophobic region. **B** The location of the FMO monomer nearest to the hydrophobic region. **B** corresponds to the eye and box shown in **A**.

**Supplementary Tables**

**Table S1.** Predicted hydrophobic thickness of representative RCs using the Positioning of Proteins in Membranes server (Lomize et al. 2012).

| **Structure** | **Thickness (Å)** |
| --- | --- |
| GsbRC (6M32) | 30.4 ± 0.6 |
| HbRC (5V8K) | 26.6 ± 0.5 |
| PSI (1JB0) | 29.8 ± 0.0 |
| PSII (3WU2) | 31.2 ± 0.5 |
| PbRC (1PRC) | 31.0 ± 0.7 |

**Table S2.** GsbRC antenna BChl site comparison with positions in the HbRC, PSI, and PSII.

| **GsbRC Site** | **HbRC** | **PSI (PsaA)** | **PSI (PsaB)** | **PSII (CP47)** | **PSII (CP43)** |
| --- | --- | --- | --- | --- | --- |
| 804* | Yes | Yes | Yes | Yes | Yes |
| 805 | Yes | Yes | Yes | Yes | Yes |
| 806 | Yes | No | No | Yes | Yes |
| 807 | Yes | Yes | Yes | Yes | Yes |
| 808 | Yes | Yes | Yes | Yes | Yes |
| 809 | Yes | Yes | Yes | Yes | Yes |
| 810 | Yes | Yes | Yes | Yes | Yes |
| 811 | Yes | Yes | Yes | No | No |
| 812 | Yes | Yes | Yes | Yes | Yes |
| 813 | Yes | Yes | Yes | Yes | Yes |
| 814 (Chl_Z/D_) | Yes | Yes | Yes | Yes | Yes |
| 815 | Yes | Yes | Yes | No | No |

*Numbers correspond to the residue number found in the PDB coordinate file.

**Supplementary References**

Chen J-H, Wu H, Xu C, et al (2020) Architecture of the photosynthetic complex from a green sulfur bacterium. Science doi: 10.1126/science.abb6350

Gisriel C, Sarrou I, Ferlez B, et al (2017) Structure of a symmetric photosynthetic reaction center-photosystem. Science doi: 10.1126/science.aan5611

Guex N, Peitsch MC, Schwede T (2009) Automated comparative protein structure modeling with SWISS-MODEL and Swiss-PdbViewer: A historical perspective. Electrophoresis 30:S162–S173. doi: 10.1002/elps.200900140

Hippler M, Drepper F (2006) Electron Transfer Between Photosystem I and Plastocyanin or Cytochrome c_6_. In: Golbeck JH (ed) Photosystem I. Advances in Photosynthesis and Respiration. Springer Netherlands, Dordrecht, pp 499–513

Jordan P, Fromme P, Witt HT, et al (2001) Three-dimensional structure of cyanobacterial Photosystem I at 2.5 Å resolution. Nature 411:909–917. doi: 10.1038/35082000

Kölsch A, Radon C, Golub M, et al (2020) Current limits of structural biology: The transient interaction between cytochrome *c*_6_ and photosystem I. Curr Res Struct Biol 2:171–179. doi: https://doi.org/10.1016/j.crstbi.2020.08.003

Kumar S, Stecher G, Tamura K (2016) MEGA7: Molecular Evolutionary Genetics Analysis version 7.0 for bigger datasets. Mol Biol Evol msw054. doi: 10.1093/molbev/msw054

Le SQ, Gascuel O (2008) An improved general amino acid replacement matrix. Mol Biol Evol 25:1307–1320. doi: 10.1093/molbev/msn067

Lomize MA, Pogozheva ID, Joo H, et al (2012) OPM database and PPM web server: resources for positioning of proteins in membranes. Nucleic Acids Res 40:D370–D376. doi: 10.1093/nar/gkr703

Pei J, Kim B-HH, Grishin N V. (2008) PROMALS3D: A tool for multiple protein sequence and structure alignments. Nucleic Acids Res 36:2295–2300. doi: 10.1093/nar/gkn072

Sievers F, Wilm A, Dineen D, et al (2011) Fast, scalable generation of high quality protein multiple sequence alignments using Clustal Omega. Mol Syst Biol 7:1–6.

Sommer F, Drepper F, Haehnel W, Hippler M (2004) The hydrophobic recognition site formed by residues PsaA-Trp^651^ and PsaB-Trp^627^ of Photosystem I in *Chlamydomonas reinhardtii* confers distinct selectivity for binding of plastocyanin and cytochrome *c*_6_. J Biol Chem 279:20009–20017. doi: 10.1074/jbc.M313986200
